# Visomitin Attenuates Pathological Bone Loss by Reprogramming Osteoclast Metabolism via the STAT3/LDHB Axis

**DOI:** 10.34133/research.0784

**Published:** 2025-07-22

**Authors:** Putao Yuan, Zhenhua Feng, Haotian Yang, Hong Xue, Hongwei Xie, Zihan Dai, Haoming Wang, Ying Liu, Bin Pan, Hongpu Song, Huali Ye, Ziang Xie, Peihua Shi, Xuewu Sun

**Affiliations:** ^1^ Department of Orthopaedic Surgery, Sir Run Run Shaw Hospital, Zhejiang University School of Medicine, Hangzhou, China.; ^2^ Zhejiang Key Laboratory of Mechanism Research and Precision Repair of Orthopaedic Trauma and Aging Diseases, Hangzhou, China.; ^3^ Department of Orthopedics, Xiangya Hospital, Central South University, Changsha, China.; ^4^ Movement System lnjury and Repair Research Center, Xiangya Hospital, Central South University, Changsha, China.; ^5^ Department of Orthopedics Surgery, The First Affiliated Hospital of Zhejiang Chinese Medical University (Zhejiang Provincial Hospital of Chinese Medicine), Hangzhou, China.; ^6^ Department of Orthopaedics, Tongde Hospital of Zhejiang Province, Hangzhou, China.

## Abstract

A persistently substantial energy demand and metabolic reprogramming endure throughout the entire course of osteoclastogenesis, accompanied by an intensified oxidative stress. Hence, balancing cellular energy metabolism and maintaining redox homeostasis offer potential for coordinating osteoclastogenesis and bone loss in pathological conditions. In the present study, we have discovered Visomitin, a novel antioxidant that specifically targets mitochondria, which efficiently decreases intracellular reactive oxygen species (ROS) levels, inhibits osteoclastogenesis, and impairs the function of bone resorption. Mechanistically, Visomitin directly targets signal transducer and activator of transcription 3 (STAT3), leading to the inhibition of its transcriptional activity and modulation of lactate dehydrogenase B (LDHB) expression levels, consequently triggering metabolic reprogramming and exerting antagonistic effects on osteoclasts. Furthermore, administration of Visomitin demonstrates marked protective effects against pathological bone loss in vivo. Given its established clinical safety profile in ophthalmologic applications, Visomitin emerges as a promising anti-resorptive agent for clinical translation. This study also unveils the STAT3/LDHB axis as a critical nexus linking mitochondrial redox regulation to osteoclast metabolism, providing a novel therapeutic strategy for osteoclast-driven bone diseases.

## Introduction

Osteoclasts, which originate from the monocyte–macrophage lineage, serve as the primary regulators of bone resorption and are essential for maintaining skeletal homeostasis [1,2]. Abnormal increases in osteoclast formation contribute to pathological bone loss observed in diseases such as osteoporosis, rheumatoid arthritis, and metastatic bone disease [3–6]. This process is tightly regulated by receptor activator of nuclear factor kappa-B ligand (RANKL) signaling, which triggers metabolic reprogramming characterized by enhanced glycolysis and oxidative phosphorylation (OxPhos), to fulfill the substantial energy requirements during osteoclast differentiation [7–11]. Mitochondria, central to both energy production and redox balance, undergo adaptive changes during osteoclastogenesis, including increased biogenesis and elevated reactive oxygen species (ROS) generation [12,13]. The excessive production of ROS disrupts redox homeostasis, further exacerbating bone resorption [14,15]. Thus, interventions coupling mitochondrial metabolism and ROS balance represent promising strategies to coordinating osteoclastogenesis and mitigating pathological bone loss.

Visomitin (SkQ1), a mitochondria-targeted antioxidant, has demonstrated therapeutic efficacy in clinical trials for dry eye syndrome (NCT03764735) and age-related pathologies [16–18]. Its decyltriphenylphosphonium (TPP^+^) moiety enables selective mitochondrial accumulation, where it scavenges ROS and modulates mitochondrial metabolism [19]. Notably, dietary Visomitin supplementation ameliorated osteoporosis in OXYS rats, a senescence-accelerated model, suggesting its unexplored potential in bone metabolism [20]. However, the mechanisms linking its antioxidant properties to osteoclast regulation remain to be elucidated. Recent studies implicate signal transducer and activator of transcription 3 (STAT3), a transcription factor (TF) activated by RANKL, in coordinating metabolic and oxidative stress responses during osteoclastogenesis [21–24]. STAT3 directly regulates the transcription of lactate dehydrogenase B (LDHB), a glycolytic enzyme critical for osteoclastogenesis and bone resorption [25,26]. Nevertheless, the exact mechanisms through which mitochondrial-targeted agents modulate this axis to influence bone resorption remain unclear.

Herein, we investigate whether Visomitin exerts anti-osteoclastogenic effects via STAT3/LDHB-mediated metabolic reprogramming. Our findings demonstrate that Visomitin directly interacts with STAT3, inhibiting its transcriptional activity and thereby suppressing LDHB expression, which disrupts glycolytic flux and lactate production while attenuating RANKL-induced oxidative stress and mitochondrial bioenergetics. This offers protection against osteoclastogenesis and pathological bone loss without compromising osteogenesis.

This study unveils a dual mechanism by which Visomitin couples redox regulation to metabolic inhibition, positioning it as a novel therapeutic candidate for osteoclast-driven bone diseases. Our findings bridge the gap between mitochondrial-targeted antioxidants and bone metabolism, offering a clinically translatable approach to mitigate excessive bone resorption.

## Results

### Application of Visomitin diminishes the intracellular ROS levels

**Fig. 1.**
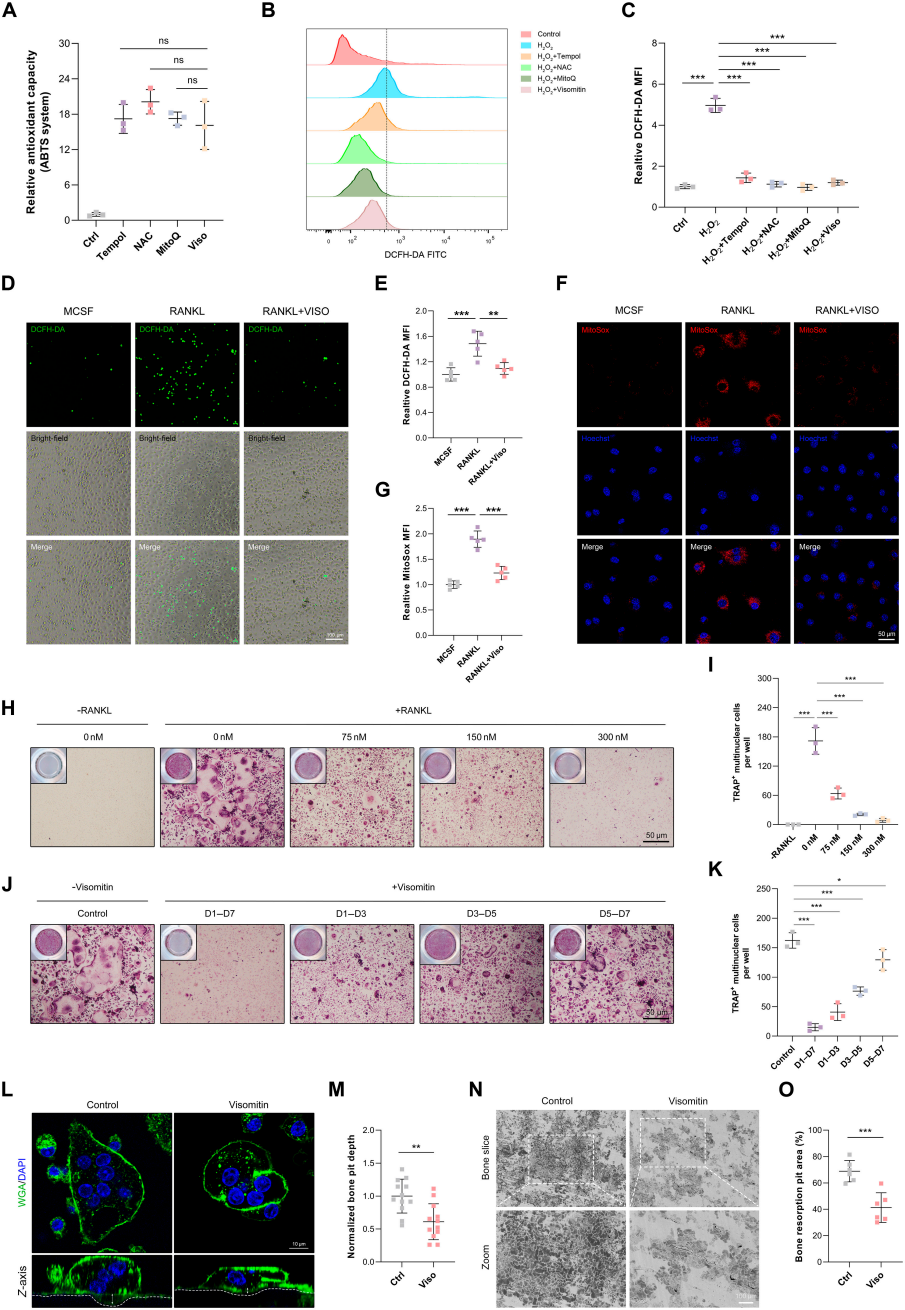
Visomitin diminishes the intracellular ROS levels and attenuates osteoclastogenesis. (A) The relative antioxidant capacity of indicated antioxidants at 300 nm was evaluated in the ABTS system (*n* = 3). (B and C) Evaluation and quantification of the intracellular ROS levels of BMMs exposed to H_2_O_2_ after pretreatment of a range of antioxidants (300 nm) using flow cytometry (*n* = 3). (D and E) Detection and quantification of the mean fluorescence intensity (MFI) of DCFH-DA probe in BMMs following treatment with either RANKL or Visomitin; scale bars, 100 μm (*n* = 5). (F and G) Detection and quantification of the MFI of MitoSOX probe in BMMs following treatment with either RANKL or Visomitin; scale bars, 50 μm (*n* = 5). (H) BMMs were treated with different dosages of Visomitin and subjected to in vitro osteoclast differentiation. Representative images of TRAP staining were shown. Scale bars, 50 μm. (I) Quantification of TRAP^+^ multinuclear cells per well in panel (A) (*n* = 3). (J) BMMs were subjected to in vitro osteoclast differentiation and treated with 300 nm Visomitin at specified stages. Representative images of TRAP staining were shown. Scale bars, 50 μm. (K) Quantification of TRAP^+^ multinuclear cells per well in panel (C) (*n* = 3). (L) Representative images of wheat germ agglutinin (WGA) staining in osteoclasts treated with or without Visomitin. Scale bars, 10 μm. (M) Quantification of bone pit depth in panel (I) (*n* = 12). (N) Representative SEM images of bone slice resorption pits. Scale bars, 10 μm. (O) Quantification of bone resorption pit area (*n* = 6). Data are mean ± SD; *P < 0.05, **P < 0.01, and ***P < 0.001; ns, not significant.

In light of Visomitin’s properties as a mitochondria-targeted antioxidant, we first evaluated its antioxidant capacity. At a concentration that did not affect the viability of bone marrow-derived macrophages (BMMs), Visomitin exhibited antioxidant capacity comparable to well-established antioxidants including Tempol, acetylcysteine (NAC), and mitoquinone mesylate (MitoQ), effectively reducing intracellular ROS levels induced by H_2_O_2_ (Fig. S1A to C and Fig. 1A to C). As osteoclast formation induced by RANKL involves a gradual increase in intracellular ROS levels [14], we also employed the 2′,7′-dichlorofluorescin diacetate (DCFH-DA) and mitochondrial superoxide (MitoSOX) probes to evaluate Visomitin’s impact on intracellular ROS levels induced by RANKL. As depicted, treatment with RANKL heightened intracellular ROS levels in BMMs, whereas Visomitin intervention significantly attenuated these tendency (Fig. 1D to G). The above data indicated that the application of Visomitin effectively mitigate the elevation of ROS in BMMs caused by external stimuli.

### Visomitin intervention suppresses osteoclastogenesis and limits bone resorption in vitro

Given Visomitin’s outstanding antioxidant properties, we conducted further evaluation of its role in osteoclastogenesis. We initially established various concentration gradients to assess the impact of Visomitin on osteoclastogenesis at different application concentrations. The results from tartrate-resistant acid phosphatase (TRAP) staining indicated that Visomitin suppressed the formation of multinucleated osteoclasts across all application concentrations, with this effect becoming increasingly pronounced as the application concentration rose (Fig. 1H and I). In addition, we conducted Visomitin interventions at various time points to assess its impact on the initial, intermediate, and final phases of osteoclast differentiation. Consistently, in all Visomitin-treated groups, the formation of multinucleated osteoclasts was inhibited to varying degrees, with the most significant impacts observed during the early and middle stages of intervention (Fig. 1J and K). Subsequently, we performed double staining on osteoclasts grown on bovine bone slices using F-actin and cathepsin K (CTSK). The findings indicated that Visomitin treatment effectively minimized the F-actin ring area and lowered the expression levels of CTSK (Fig. S1D to G). Furthermore, the expression of osteoclast-related markers was also suppressed by Visomitin, as confirmed by Western blot (WB) and quantitative real-time polymerase chain reaction (RT-qPCR) analyses (Fig. S1H to K). Fully differentiated multinucleated osteoclasts adhere to the bone surface and secrete protons along with lysosomal hydrolases, enabling their role in bone resorption [27]. Therefore, we further examined the effect of Visomitin on the bone resorption activity of osteoclasts. Our findings indicated that the application of Visomitin to fully differentiated osteoclasts significantly attenuated their bone resorption capacity, as evidenced by a reduction in both the depth and area of bone resorption pits (Fig. 1L to O). Collectively, these results demonstrate that Visomitin inhibits osteoclast formation and suppresses their bone resorption activity in vitro.

### The administration of Visomitin confers a protective effect against pathological bone loss in vivo

**Fig. 2.**
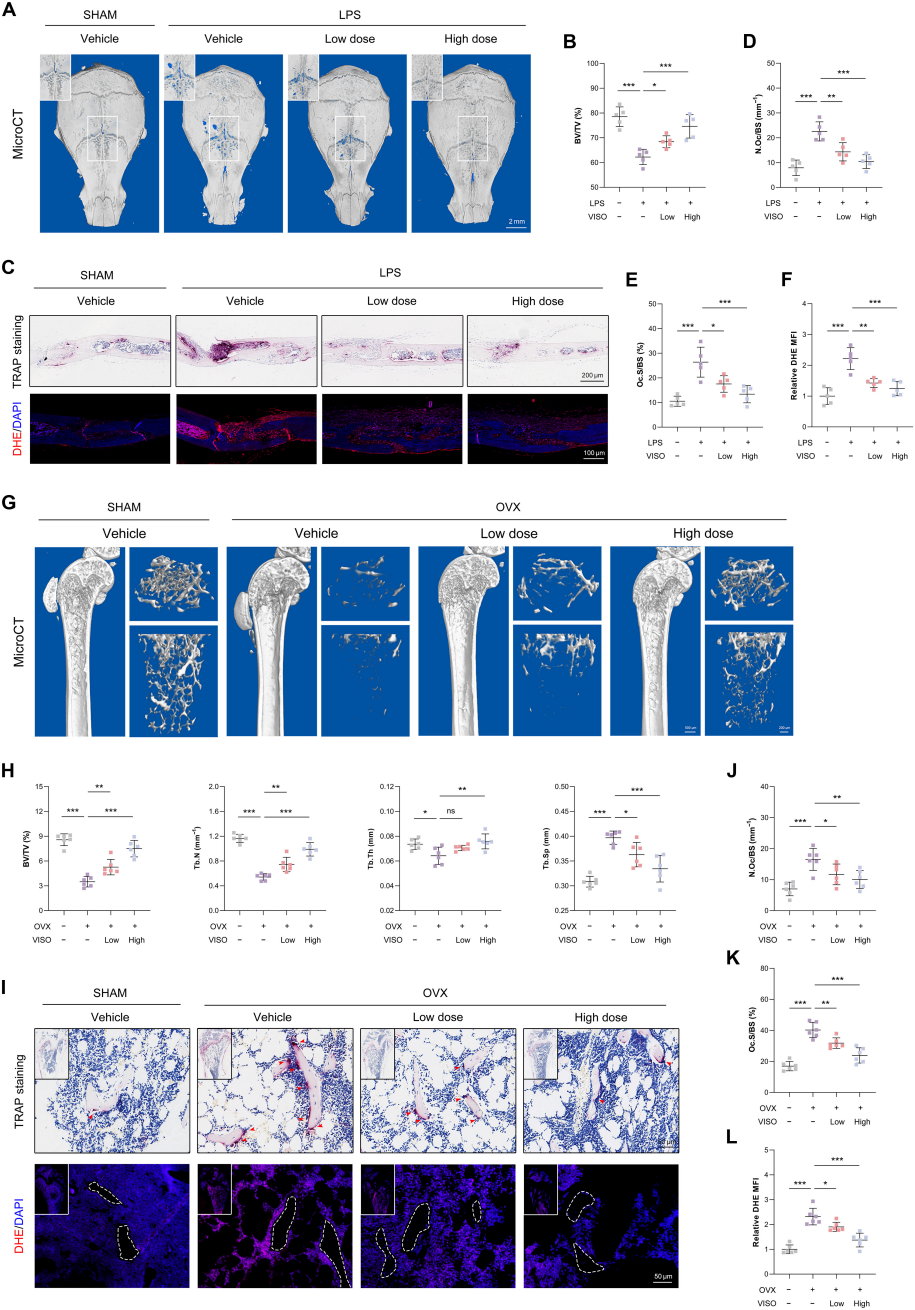
Administration of Visomitin alleviates pathological bone loss in vivo. (A) Representative 3D micro-CT images of the calvaria from mice subjected to either sham or LPS injection, followed by treatment with PBS or Visomitin. Scale bars, 2 mm. (B) Quantification of BV/TV (%) in panel (A) (*n* = 5). (C) Representative TRAP and DHE staining of the calvaria from designated groups. Scale bars, 200 μm and 100 μm, respectively. (D to F) Quantification of N.Oc/BS (mm^−1^), Oc.S/BS (%), and relative DHE MFI in panel (C) (*n* = 5). (G) Representative 3D micro-CT images of the femurs from mice subjected to either sham or OVX operation, followed by treatment with PBS or Visomitin. Scale bars, 500 and 200 mm, respectively. (H) Quantification of BV/TV (%), Tb.N (mm^−1^), Tb.Th (mm), and Tb.Sp (mm) in panel (G) (*n* = 6). (I) Representative TRAP and DHE staining of the femurs from designated groups. Scale bars, 50 μm. (J to L) Quantification of N.Oc/BS (mm^−1^), Oc.S/BS (%), and relative DHE MFI in panel (I) (*n* = 6).Data are mean ±SD; *P < 0.05, **P < 0.01, and ***P < 0.001; ns, not significant.

Given the pronounced osteoclast-antagonistic effect of Visomitin observed in vitro, we subsequently established 2 pathological animal models to assess its protective effects in vivo. In an osteolysis model, supplementation with various doses of Visomitin effectively ameliorated local bone erosion in the mouse skull, as indicated by micro-CT results demonstrating increased trabecular bone volume per total volume (BV/TV) ratio compared to that observed in the LPS + Vehicle group (Fig. 2A and B). Subsequent histological staining of the cranial sections revealed that Visomitin application resulted in a marked decrease in the number of osteoclasts within the local lesion area, which, in turn, helped to alleviate pathological bone loss (Fig. S2A and C to E). Additionally, dihydroethidium (DHE) staining demonstrated that Visomitin usage could attenuate local peroxide production (Fig. 2C and F). Similar outcomes were also observed in an ovariectomy (OVX)-induced osteoporosis model. Daily administration of Visomitin effectively ameliorated estrogen deficiency-induced bone loss in mice, as manifested by varying degrees of up-regulation for micro-CT parameters including BV/TV, trabecular number (Tb.N), and trabecular thickness (Tb.Th) compared with the OVX + Vehicle group; meanwhile, an adaptive down-regulation is observed in the trabecular separation (Tb.Sp) parameter (Fig. 2G and H). Consistently, the quantity of osteoclasts and the corresponding area were diminished following treatment with Visomitin, as demonstrated by TRAP staining (Fig. S2B and Fig. 2I to K). Through DHE staining of femoral sections, we further validated that the administration of Visomitin effectively improves the redox balance in mice and reduces tissue peroxide levels (Fig. 2I and L).

Given the lack of osteoclast-targeting properties in routine Visomitin supplementation, we conducted an in-depth investigation into its potential effects on osteogenesis, another critical component for maintaining skeletal homeostasis [28,29]. In contrast to serum CTXI levels, serum P1NP levels, which serve as an indicator of osteogenic activity, showed no significant variation between the Visomitin-treated group and the Vehicle-treated group (Fig. S2C and D). Furthermore, calcein double labeling, in conjunction with immunohistochemical staining for osteocalcin (OCN) on femoral sections, revealed that in mice administered Visomitin, there were no discernible differences in mineral apposition rate (MAR) and OCN-positive osteoblasts compared to the Vehicle group, suggesting that Visomitin exerts a minimal impact on bone formation in vivo (Fig. S2E to H). Moreover, hematoxylin–eosin (HE) staining of internal organs and detection of serum biochemical indicators in mice from different groups revealed that supplementation with Visomitin did not induce any discernible toxic effects, indicating its favorable safety profile (Fig. S3A and B). Following this, murine cranial osteoprogenitor cells were isolated to evaluate the in vitro influence of Visomitin on osteogenesis. The CCK8 assay demonstrated that the use of Visomitin at an equivalent concentration to BMMs did not exert any impact on the cell viability of osteoprogenitor cells (Fig. S4A and B). Furthermore, the results of alkaline phosphatase (ALP) staining combined with Alizarin Red S (ARS) staining demonstrated that applying different concentrations of Visomitin in vitro, consistent with its dosage in BMMs, does not affect osteoblast differentiation and mineralization (Fig. S4C). WB along with quantitative real-time polymerase chain reaction (RT-qPCR) assays revealed no significant alteration in the expression of osteoblast-specific markers following intervention with varying concentrations of Visomitin as well (Fig. S4D and E). These results suggest that Visomitin administration confers a protective effect against pathological bone loss in vivo and mitigates oxidative stress without impacting osteogenesis.

### Transcriptomic RNA sequencing of osteoclasts treated with Visomitin

**Fig. 3.**
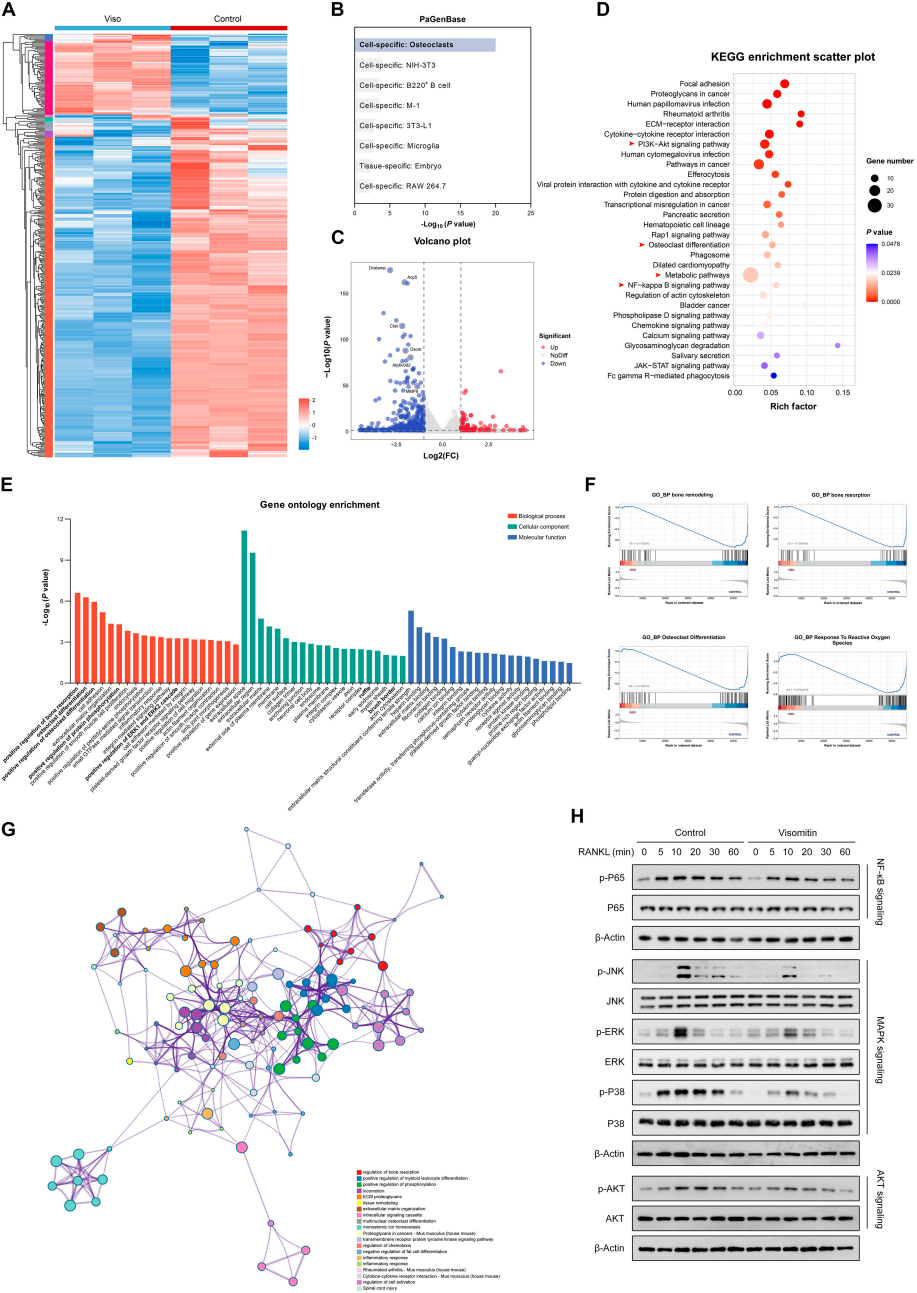
Visomitin attenuates the activation of RANKL-RANK signaling pathways. BMMs treated with Visomitin or PBS were subjected to osteoclast differentiation, followed by Transcriptome RNA-seq. Genes with |log_2_FC| > 1, *P* < 0.05, and TPM > 0.5 are designated as differentially expressed genes (DEGs) (*n* = 3). (A) The heatmap illustrating the gene expression profiles derived from RNA-seq data. (B) Analysis of RNA-seq data for cell and tissue specificity utilizing the PaGenBase database. (C) The volcano plot illustrating the gene expression profiles derived from RNA-seq data. (D) KEGG enrichment analysis of DEGs obtained from RNA-seq data. (E) GO enrichment analysis of down-regulated genes (Log_2_FC < −1 and *P* < 0.05) obtained from RNA-seq data. (F) GSEA of Gene Ontology Biological Processes in RNA-seq data. (G) Network of enrich terms derived from RNA-seq data utilizing the Metascape database. (H) Representative immunoblots illustrating the effects of Visomitin on the activation of RANKL-RANK signaling pathways, including NF-κB, MAPK, and AKT pathways (*n* = 3). Data are mean ± SD; *P < 0.05, **P < 0.01, and ***P < 0.001; ns, not significant.

To gain deeper insights into the mechanisms by which Visomitin regulates osteoclastogenesis, BMMs treated with either Visomitin or phosphate-buffered saline (PBS) were subjected to in vitro osteoclast differentiation and subsequently underwent transcriptome RNA sequencing. Genes with a |Log_2_FC| of at least 1 and a *P* value below 0.05 were identified as differentially expressed genes (DEGs). The results were visualized in the form of a heatmap (Fig. 3A). The cell specificity was validated using the PaGenBase database, and the distribution of all genes was illustrated in a volcano plot (Fig. 3B and C). Notably, key osteoclast-specific markers including Dcstamp, Ctsk, Oscar, and Acp5 showed significant down-regulation upon Visomitin treatment, reinforcing the idea that Visomitin effectively inhibits osteoclast formation. Further Kyoto Encyclopedia of Genes and Genomes (KEGG) pathway enrichment analysis of the DEGs indicated that these genes were primarily linked to the phosphatidylinositol-3-kinase/Akt (PI3K-Akt) signaling pathway, osteoclast differentiation, metabolic pathways, and nuclear factor kappa-light-chain-enhancer of activated B cells (NF-kB) signaling pathways (Fig. 3D). The Gene Ontology (GO) enrichment analysis of down-regulated DEGs also revealed an enrichment of pathways such as osteoclast differentiation and positive regulation of protein phosphorylation (Fig. 3E). Likewise, the Gene Set Enrichment Analysis (GSEA) demonstrated a significant enrichment of pathways associated with osteoclast differentiation, response to ROS, etc., consistent with the previously mentioned findings (Fig. 3F). We also constructed a network of enriched terms, as illustrated in Fig. 3G. Given the enrichment analyses from both KEGG and GO, which indicated that DEGs were associated with multiple RANKL-RANK signaling pathways, we further validated these findings through WB assay. The findings revealed different levels of suppression in the NF-κB, mitogen-activated protein kinases (MAPKs), and serine/threonine protein kinase (AKT) signalings after Visomitin treatment (Fig. 3H). In light of the aforementioned results, it can be further inferred that Visomitin has a suppressive effect on osteoclastogenesis and the RANKL-RANK signaling pathway.

### Visomitin treatment induces metabolic reprogramming during osteoclastogenesis

**Fig. 4.**
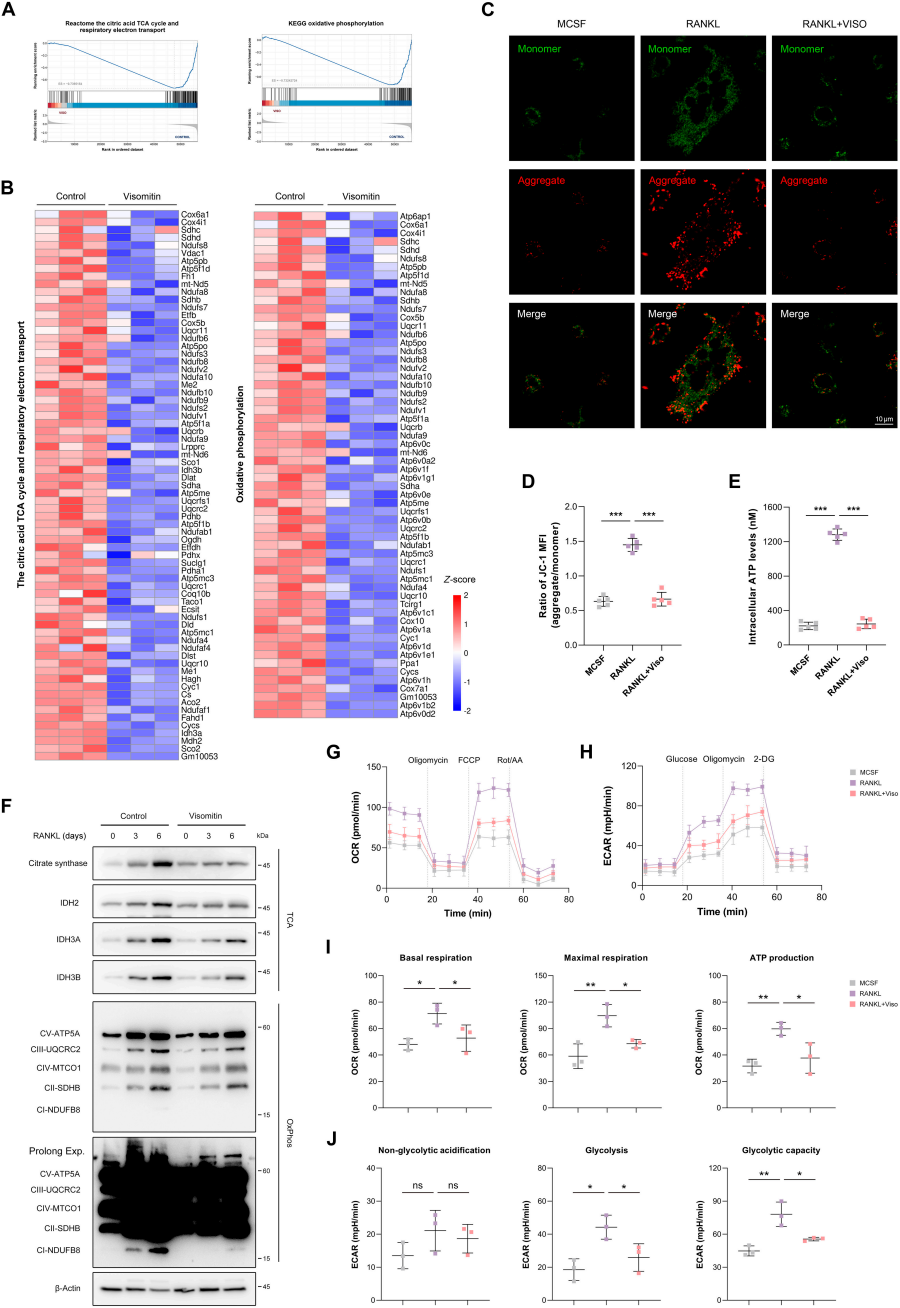
Visomitin reprograms energy metabolism during osteoclastogenesis. (A) GSEA analysis of Reactome and KEGG pathways in RNA-seq data. (B) The heatmap depicting the gene expression profiles of the 2 pathways presented in panel (A). (C) Representative JC-1 staining of BMMs treated with MCSF, RANKL, or Visomitin as indicated. (D) Quantification of JC-1 staining as presented in panel (C) (*n* = 5). (E) Intracellular ATP levels following treatment with MCSF, RANKL, or Visomitin as indicated (*n* = 5). (F) Representative immunoblots illustrating the expression levels of proteins within the aforementioned 2 pathways following Visomitin treatment (*n* = 3). (G and H) Representative Seahorse graphs for oxygen consumption rate (OCR) or extracellular acidification rate (ECAR) (*n* = 3). (I) Quantification of the basal respiration, maximal respiration, and ATP production from OCR data. (J) Quantification of the non-glycolytic acidification, glycolysis, and glycolytic capacity from ECAR data. Data are mean ± SD; *P < 0.05, **P < 0.01, and ***P < 0.001; ns, not significant.

According to the GSEA, Visomitin treatment is closely linked to The Citric Acid (TCA) Cycle and Respiratory Electron Transport, along with Oxidative Phosphorylation pathways (Fig. 4A). Consequently, we integrated the DEGs from both pathways and visualized them in a heatmap format (Fig. 4B). Given Visomitin’s properties to target mitochondria and the close relationship between mitochondria and cellular energy metabolism, we utilized JC-1 probe to evaluate mitochondrial membrane potential in differentially treated cells. Our results presented that RANKL treatment elevated intracellular mitochondrial membrane potential, while Visomitin intervention mitigated this effect (Fig. 4C and D). Additionally, Visomitin treatment significantly inhibited adenosine triphosphate (ATP) production in RANKL-stimulated cells (Fig. 4E). Subsequently, we further accessed the expression levels of TCA cycle and oxidative phosphorylation-related proteins through WB. Consistent with the transcriptome results, Visomitin treatment attenuated the expression levels of these proteins to varying extents (Fig. 4F). The Seahorse assays further confirmed that Visomitin intervention effectively inhibits energy metabolism levels during the process of osteoclastogenesis (Fig. 4G to J). Collectively, these findings indicate that the intervention with Visomitin has the potential to reprogram energy metabolism during osteoclastogenesis, leading to an osteoclast-antagonistic effect.

### Visomitin exerts a negative regulatory effect on LDHB expression and lactate production

**Fig. 5.**
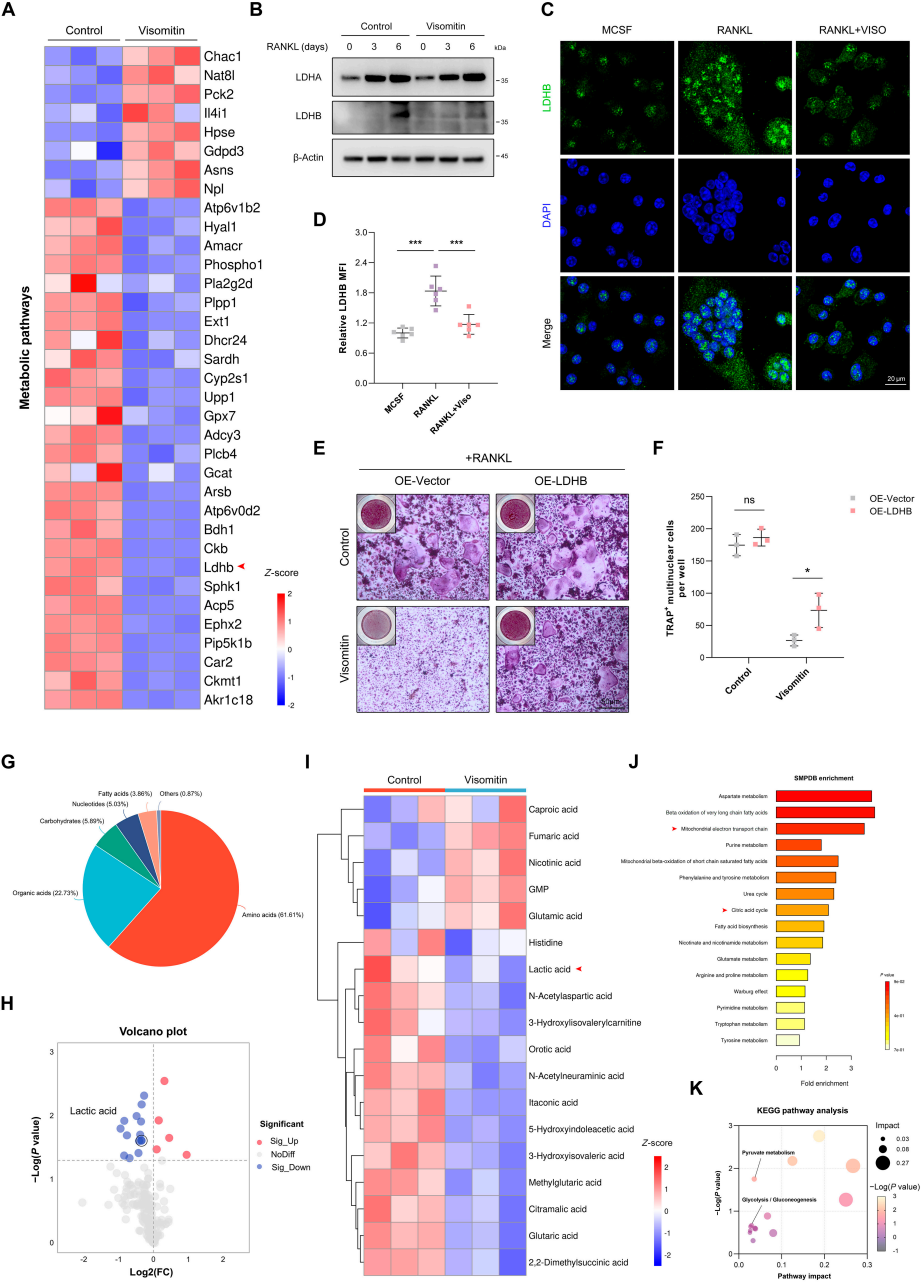
Visomitin regulates osteoclastogenesis through the LDHB–lactate axis. (A) The heatmap depicting the gene expression profiles of metabolic pathways. (B) Representative immunoblots depicting the protein levels of LDHA and LDHB following Visomitin treatment (*n* = 3). (C) Immunofluorescence staining of LDHB in BMMs subjected to osteoclast differentiation, with or without Visomitin treatment; scale bars, 20 μm. (D) Quantification of the relative LDHB MFI in panel (C) (*n* = 6). (E) BMMs infected with either the vector or LDHB-overexpressing adenovirus were differentiated into osteoclasts in the presence or absence of Visomitin treatment. Representative images of TRAP staining were shown. Scale bars, 50 μm. (F) Quantification of TRAP^+^ multinuclear cells per well in panel (E) (*n* = 3). (G) The classification of metabolites detected through metabolomics. (H and I) The volcano plot and heatmap illustrating the metabolite affinity profiles derived from metabolomics. (J and K) Enrichment analysis of differential metabolites detected by metabolomics using SMPDB and KEGG databases. Data are mean ± SD; *P < 0.05, **P < 0.01, and ***P < 0.001; ns, not significant.

Considering the findings from the previous KEGG enrichment analysis, which indicated a significant association between Visomitin and key genes in metabolic pathways, as well as its ability to reprogram metabolism during osteoclastogenesis, we illustrate the expression patterns of metabolism-related genes using a heatmap format to more precisely identify the downstream target genes associated with the effects of Visomitin (Fig. 5A). Notably, LDHB, a critical effector molecule in glycolysis, was significantly down-regulated in the Visomitin-treated group. Acting as isoenzymes, LDHB and LDHA form either homo- or hetero-tetramers of lactate dehydrogenase (LDH), enabling the interconversion between pyruvate and lactate [30]. Earlier research has shown that knocking out LDHB can substantially suppress osteoclast differentiation, along with a reduction in extracellular acidity, which, in turn, inhibits bone resorption [26]. Consequently, we further assessed the expression of LDHB following Visomitin treatment using WB and immunofluorescence, which revealed that Visomitin treatment significantly reduced LDHB expression during osteoclast differentiation while exerting minimal effects on LDHA levels (Fig. 5B to D). Additionally, overexpression of LDHB in BMMs partially mitigated the inhibitory effect of Visomitin on osteoclast differentiation (Fig. 5E and F and Fig. S5A and B). To further elucidate the regulatory role of Visomitin on metabolic processes during osteoclast differentiation, we performed Q300 quantitative metabolomics analysis; this yielded results consistent with previous findings indicating that Visomitin modulates relevant metabolite levels during osteoclast differentiation and inhibits lactate production (Fig. 5G to I and Fig. S6A to C). Small Molecule Pathway Database‌ (SMPDB) and KEGG enrichment analyses indicated that intervention with Visomitin effectively influences pathways related to Mitochondrial Electron Transport Chain, Tricarboxylic Acid Cycle, Pyruvate Metabolism, and Glycolysis (Fig. 5J and K). These findings further elucidate that Visomitin modulates LDHB expression to trigger metabolic reprogramming and exerting antagonistic effects on osteoclasts.

### STAT3 serves as a direct target of Visomitin and is implicated in the transcriptional regulation of LDHB

**Fig. 6.**
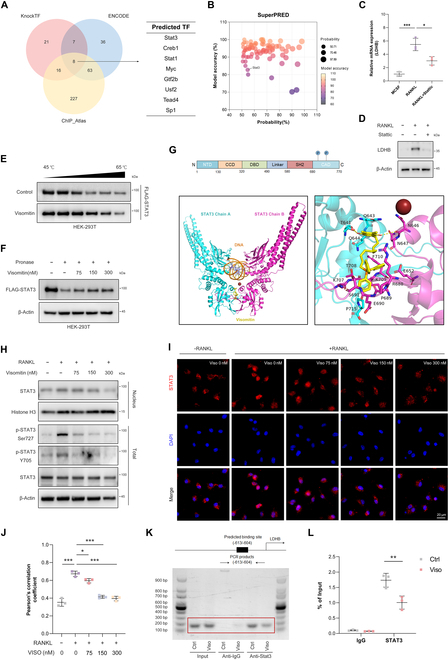
STAT3 functions as a direct target of Visomitin to modulate LDHB transcription. (A) The potential transcription factors (TFs) for LDHB were predicted using the KnockTF, ENCODE, and ChIP_Atlas databases. (B) The potential targets of Visomitin were predicted using the SuperPRED database. (C and D) The mRNA and protein expression levels of LDHB under Stattic treatment (*n* = 3). (E) The thermal stability of FLAG-STAT3 under Visomitin treatment was detected using WB (*n* = 3). (F) The stability of FLAG-STAT3 in the presence of protease following treatment with Visomitin (0, 75, 150, and 300 nmol/l) was detected using WB (*n* = 3). (G) Three-dimensional image of molecular docking between Visomitin and STAT3. (H) Representative immunoblots for the indicated nuclear, phosphorylated, or total proteins following treatment with Visomitin (0, 75, 150, and 300 nm) (*n* = 3). (I) Representative Immunofluorescence images of STAT3 in BMMs after treatment with RANKL or Visomitin (0, 75, 150, and 300 nm) as indicated. Scale bars, 20 μm. (J) Quantification of Pearson’s correlation coefficient between STAT3 and DAPI in panel (I) (*n* = 3). (K) STAT3 ChIP assay of LDHB promoter region. (L) Quantification of the binding affinity between STAT3 and the LDHB promoter (*n* = 3). Data are mean ± SD; *P < 0.05, **P < 0.01, and ***P < 0.001; ns, not significant.

Given the impact of Visomitin intervention on LDHB transcriptional expression, we utilized 3 public databases to predict potential TFs of LDHB. As illustrated in Fig. 6A, 8 TFs were identified as overlapping across the 3 databases. Among these, only STAT3 was predicted as a potential target of Visomitin based on the results from the SuperPRED database (Fig. 6B). Given STAT3’s pivotal role in skeletal homeostasis and the transcriptional regulation of LDHB, we further explored the potential of Visomitin to modulate LDHB transcription via STAT3 [25,31]. Stattic, a selective inhibitor of STAT3, was subsequently incorporated into the cell culture system. It was observed that its addition significantly suppressed RANKL-induced LDHB expression (Fig. 6C and D). Additionally, activation of STAT3 by Colivelin mitigated the inhibitory effect of Visomitin on LDHB (Fig. S7A and B). Subsequent drug affinity responsive target stability (DARTS) and cellular thermal shift assay (CETSA) experiments further provide evidence that Visomitin treatment significantly reduces the degradation of FLAG-STAT3 protein induced by elevated temperature and protease addition, thereby confirming the direct targeting of STAT3 by Visomitin (Fig. 6E and F). Consequently, we employed molecular docking to simulate the binding mode between Visomitin and STAT3. The simulation findings suggest that the ligand Visomitin is capable of binding to the SH3 domain of the STAT3 protein, with a favorable binding energy of −6.956 kcal/mol (Fig. 6G). The SH2 domain, serving as the primary effector domain of STAT3, plays a crucial role in mediating STAT3 nuclear translocation and transcriptional activity [32–34]. Moreover, it represents a direct target for numerous selective inhibitors of STAT3 [35]. We therefore conducted a separate analysis of nuclear protein detection to explore the impact of Visomitin intervention on STAT3 nuclear translocation. The WB assay revealed a significant inhibition of the intranuclear transport of STAT3 by Visomitin, as well as its negative regulatory impact on the total protein phosphorylation of STAT3 (Fig. 6H). The immunofluorescence assays yielded similar results, demonstrating that Visomitin intervention significantly attenuated RANKL-induced nuclear translocation of STAT3 (Fig. 6I and J). Moreover, the CHIP assay data also revealed a reduction in the binding affinity of STAT3 with the LDHB promoter in the presence of Visomitin, suggesting a novel regulatory pattern involving the Visomitin–STAT3–LDHB axis (Fig. 6K and L). In conclusion, STAT3 acts as a directed target of Visomitin and participates in the transcriptional regulation of LDHB, thus influencing osteoclast formation via metabolic reprogramming.

## Discussion

Oxidation-reduction (redox) reactions are essential biochemical processes that underpin all forms of life [36–38]. Disruption of redox homeostasis, coupled with increased ROS levels, has been associated with various pathological conditions, including the promotion of osteoclastogenesis and heightened bone resorption [14,39,40]. Consequently, redox modulation is increasingly recognized as a promising therapeutic approach for the regulation of osteoclastogenesis. This study elucidates a previously unrecognized mechanism by which Visomitin, a mitochondria-targeted antioxidant, suppresses osteoclastogenesis via STAT3/LDHB-mediated metabolic reprogramming. Intervention with Visomitin significantly mitigated pathological bone loss in mouse models of osteolysis and OVX, thereby providing compelling evidence for its therapeutic potential.

Referred to as "the powerhouse of the cell", mitochondria act as a fundamental element in energy metabolism and redox regulation, covering aspects ranging from cristae dynamics to interorganellar crosstalk [41,42]. During the formation of multinucleated osteoclasts, a considerable energy demand is typically manifested, accompanied by an adaptive increase in mitochondrial quantity and a significant elevation in intracellular ROS levels, which has been demonstrated to further stimulate osteoclastogenesis [12–14]. Thus, the mitochondria targeting alongside the antioxidant properties of Visomitin offers a promising avenue for the modulation of osteoclastogenesis. Preliminary assessments of Visomitin’s antioxidant capacity reveal that, at equivalent concentrations, its efficacy is comparable to that of several established antioxidants. Incubation with Visomitin markedly attenuates the RANKL-induced increase in intracellular ROS and mitigates osteoclastogenesis with dosage dependence, exerting a pronounced effect during the early stages of osteoclastogenesis. Upon maturation, multinucleated osteoclasts facilitate the resorption of local osteal tissue through the secretion of acids and proteolytic enzymes [27,43,44]. This resorption process is also diminished in the presence of Visomitin. The aforementioned in vitro phenotypes were further validated using 2 pathological models. Furthermore, unlike bisphosphonates, which impair osteogenesis at high doses [45]. Visomitin selectively targets osteoclast without affecting bone formation, as evidenced by unchanged P1NP levels and osteocalcin staining in vivo, underscoring its significant therapeutic potential for conditions associated with pathological bone resorption.

The complex process of osteoclastogenesis is governed by a finely tuned network involving multiple regulatory factors. Among these, M-CSF plays a critical role in supporting the survival and proliferation of precursor cells, while RANKL acts as the primary trigger and sustainer of differentiation [1,44]. Precise coordination within the RANKL-RANK signaling cascades is pivotal in maintaining delicate balance in osteoclast differentiation and skeletal homeostasis. Through the integration of KEGG and GO enrichment analyses of RNA sequencing (RNA-seq) data, we observed that Visomitin significantly inhibited the expression of genes associated with osteoclast while markedly influencing the abundance of pertinent molecules within the RANKL-RANK signaling cascades. The activation of MAPK, NF-kB, and AKT signaling pathways in response to RANKL stimulation has been further demonstrated to be attenuated by Visomitin, albeit to varying extents. Given that ROS are not only essential for daily oxidation-reduction reactions but also serve as intracellular second messengers that facilitate the integration and modulation of intricate signaling pathways [46], it is plausible to assert that the antioxidant properties of Visomitin, which can effectively down-regulate ROS levels within cells, provide a compelling basis for its inhibitory effect on the RANKL-RANK signaling pathway. Notably, unlike antioxidants such as Tussilagone and Alpinetin, which exert their anti-osteoclastogenic effects by modulating the kelch-like ECH-associated protein 1 (KEAP1) /nuclear factor erythroid 2-related factor 2 (NRF2) pathway to combat oxidative stress [47,48], the antioxidant property of Visomitin is likely independent of the KEAP1/NRF2 system and instead be mediated by directly targeting mitochondria for ROS elimination. This unique antioxidant mechanism of Visomitin will also constitute one of the pivotal focus for our future research endeavors.

Osteoclast differentiation is an energy-intensive process that relies concurrently on the TCA cycle and glycolysis to fulfill its energy and biosynthetic requirements [49,50]. The TCA cycle supplies reducing equivalents (NADH and FADH2) to the electron transport chain, thereby enabling efficient ATP production, whereas glycolysis not only rapidly generates ATP but also provides essential intermediate metabolites that support lysosomal acidification [50]. This metabolic flexibility enables osteoclasts to maintain their functionality across a range of microenvironments, including hypoxic or nutrient-limited conditions. Consequently, inhibiting a single metabolic pathway may be insufficient to fully block osteoclastogenesis. By employing JC-1 staining, WB, Seahorse assays, and metabolomics, we identified that Visomitin exerts concurrent inhibitory effects on both the TCA cycle and glycolysis. This dual inhibition may underlie its potent suppression of osteoclastogenesis. Besides, lactate, a critical by-product of glycolysis, exhibits a marked reduction following Visomitin treatment. As documented, variations in lactate levels influence the acidification of intracellular and extracellular environments, which has been shown to be closely linked to the bone resorption process [26,51,52]. Consequently, the observed reduction in lactate concentration in Visomitin-treated cells provides partial clarification of the previously noted limitation on the bone resorption capacity of mature osteoclasts when exposed to Visomitin, thereby providing theoretical support for its dual regulatory role in the independent processes of osteoclast differentiation and bone resorption.

LDH serves as a pivotal enzyme within the glycolytic pathway, facilitating the reversible interconversion between lactate and pyruvate [53]. The enzyme is composed of subunits including LDHA, LDHB, and LDHC, of which LDHA and LDHB are the most prevalent [30,54]. Research indicates that LDHA demonstrates a higher affinity for pyruvate, whereas LDHB shows a more significant preference for lactate. In mammalian organisms, LDH is composed of 2 subunits—LDHA and LDHB—that combine to form a tetramer, resulting in the production of 5 isoenzymes with distinct tissue distributions [30]. In terms of osteoclast precursors and mature osteoclasts, LDHA_2_B_2_ represents the isoenzyme with the highest abundance, whereas LDHA_1_B_3_ and LDHB_4_ also exhibit increased levels during osteoclastogenesis [26]. Our findings identified that the LDHB expression is significantly reduced in the Visomitin group, which may directly constrain the activity of the LDH in osteoclast precursor cells, thereby inhibiting glycolysis and energy metabolism while negatively regulating osteoclast differentiation. Furthermore, the overexpression of LDHB in BMMs partially counteracts the osteoclast-inhibitory effect of Visomitin, thereby providing additional support to the notion that metabolic reprogramming related to LDHB contributes to Visomitin’s inhibitory action on osteoclastogenesis. Notably, overexpression of LDHB alone did not exert a significant impact on osteoclast differentiation, potentially due to the constrained abundance of the predominant isoenzyme LDHA_2_B_2_, which is limited by the expression level of LDHA. Moreover, the distinct roles of various LDH isoenzymes in osteoclast differentiation represent a critical issue that warrants further investigation in future research.

In light of the influence of Visomitin on LDHB transcription, we identified potential TFs that may regulate LDHB. Among these predicted TFs, STAT3 was also listed as a target of Visomitin based on the predictions from the SupreBRED database and has been proven to regulate LDHB transcription [25,55]. DARTS, in conjunction with CETSA assays, further substantiates that STAT3 acts as a direct target of Visomitin, with treatment resulting in the inhibition of both its phosphorylation and nuclear translocation, processes that have been shown to be linked to its transcriptional activity [32–34]. This modulation consequently imposed a negative regulatory effect on LDHB transcription. Regrettably, the inherent limitations of Visomitin’s chemical structure precluded us from conjugating biotin to its molecular framework. This constraint hindered our ability to employ co-immunoprecipitation and mass spectrometry analyses for validating the direct interaction between Visomitin and STAT3. Besides, the specific domain of STAT3 that interacts with Visomitin could only be delineated through molecular docking, representing another limitation of this study. Moreover, although Visomitin’s safety profile has already been established in Phase III clinical trials for dry eye syndrome and no significant toxic effects were observed in our murine models, long-term studies are necessary to evaluate the potential off-target effects of STAT3 inhibition, considering the pleiotropic roles of STAT3 in immunity and hematopoiesis.

In conclusion, this study establishes Visomitin as a dual regulator of redox and metabolic homeostasis in osteoclasts, offering therapeutic potential for pathological bone loss. By linking mitochondrial targeting to transcriptional control of metabolism, our findings provide a framework for repurposing redox-active compounds in bone homeostasis.

## Materials and Methods

### Reagents

Visomitin (Cat. No. HY-100474) and Calcein (Cat. No. HY-D0040) were procured from MedChemExpress (MCE). Fetal bovine serum (FBS) was sourced from CellMax. Alpha-Minimum Essential Medium (α-MEM) and Dulbecco’s Modified Eagle Medium (DMEM) were supplied by Procell. Trypsin-EDTA solution (0.25%) and Penicillin–Streptomycin Liquid (100×) were procured from Solarbio. Recombinant soluble mouse macrophage colony-stimulating factor (M-CSF) (Cat. No. CB34) was supplied by Novoprotein; Recombinant soluble mouse RANKL (Cat. No. 462-TEC-010) were purchased from R&D Systems; Total Antioxidant Capacity (T-AOC) Assay Kit (Cat. No. S0121), ROS Assay Kit (Cat. No. S0033S), Mitochondrial membrane potential assay kit with JC-1 (Cat. No. C2006), MitoSOX Assay Kit (Cat. No. S0061S), DHE (Cat. No. S0063), and Antifade Mounting Medium (Cat. No. P0126) were supplied by Beyotime. iF488-wheat germ agglutinin (Cat. No. G1730) was purchased from Servicebio. Detailed descriptions of the specific reagents are provided in the corresponding methodologies section, and Table S1 lists the antibodies used in this study.

### Mice

Wild-type mice with a pure C57BL/6 background were procured from Hangzhou Qizhen Experimental Animal Technology. The Laboratory Animal Center of Run Run Shaw Hospital provided the specific pathogen-free environment for the mice housing. These comprised a 12-h light/dark cycle, a temperature range of 22 to 24 °C, and unrestricted access to food and water. All animal experiments were conducted under the approval of the Ethics Committee of Sir Run Run Shaw Hospital, Zhejiang University School of Medicine.

### Cell culture

Isolation of BMMs: BMMs were isolated using a method previously described [56]. In summary, the tibiae and femora of 6-week-old mice were collected, and the bone marrow cavity was subsequently flushed with a 1-ml syringe to harvest bone marrow cells. The collected cells were subjected to erythrocyte lysis and then cultured in α-MEM containing 25 ng/ml mouse M-CSF for 4 days. For the osteoclastic differentiation experiment, BMMs were plated at an optimal density and stimulated with conditioned medium prepared using α-MEM supplemented with 25 ng/ml M-CSF and 50 ng/ml RANKL for 5 or 6 days until mature multinucleated osteoclasts formed.

Isolation of osteoprogenitor cells: Osteoprogenitor cells were isolated following a previously established protocol [57]. Briefly, collect the cranial bones from 3-day-old C57BL/6 mouse pups, remove any excess soft tissue and periosteum, and fragment them into small pieces prior to overnight digestion with type II collagenase. The following day, filter the cell suspension obtained from digestion and seed it in α-MEM, then culture in a sterile incubator at 37 °C with 5% CO_2_ to generate osteoprogenitor cells. For the osteogenic differentiation assay, the obtained osteoprogenitor cells were cultured at an optimal density and exposed to a conditioned medium derived from α-MEM containing 50 μM ascorbic acid, 10 mM β-glycerophosphate, and 100 nM dexamethasone for either 7 or 21 days. This was followed by staining with ALP and ARS to evaluate the extent of differentiation and mineralization.

HEK-293T cells were acquired from the American Type Culture Collection and maintained in DMEM complete medium. The authenticity of the cell line was verified by short tandem repeat (STR) profiling.

### Cell viability assay

The impact of Visomitin on the survival of BMMs and osteoprogenitor cells was evaluated using the CCK-8 assay kit (HY-K0301, MCE, USA). In summary, cells were plated in 96-well plates at a density of 1.5 × 10^4^ cells per well and maintained in medium containing various concentrations of Visomitin for either 48 or 96 h. Following this, the medium was exchanged with 10% CCK-8 solution for an additional 2 h of incubation. The absorbance in each well was then measured at 450 nm using an ELX800 absorbance microplate reader (BioTek Instruments, Winooski, VT, USA) to determine the relative cell viability after exposure to different concentrations of Visomitin.

### T-AOC assay

The assessment of T-AOC was carried out employing a T-AOC Assay Kit (S0121, Beyotime, China). In brief, the cells treated under experimental conditions were collected and fully lysed by sonication in ice-cold PBS buffer. Subsequently, the samples were centrifuged at 12,000 *g* for 5 min at 4 °C to obtain the supernatants. A 96-well plate was prepared, into which 20 μl of peroxidase working solution, 170 μl of ABTS working solution, and 10 μl of supernatants from various treatments were added to each well. Additionally, 10 μl of PBS was included as a blank control, along with varying concentrations of Trolox standard solution for the construction of the standard curve. After a 6-min incubation at room temperature, absorbance was measured at 414 nm using an ELX800 absorbance microplate reader for the calculation of relative antioxidant capacity.

### ROS assay

The ROS levels were measured using the ROS Assay Kit (S0033S, Beyotime, China). For flow cytometry analysis, the DCFH-DA probe was prepared at a working concentration of 10 μmol/l in serum-free culture medium. Pretreated BMMs were harvested, resuspended in the working solution, and incubated following the instructions. Following this, the cells were rinsed with PBS to ensure complete removal of any unincorporated DCFH-DA before flow cytometry analysis. For fluorescence analysis, BMMs were plated at an appropriate density and exposed to different treatment conditions. The culture medium was replaced with 10 μmol/l DCFH-DA working solution, and the cells were incubated. Afterward, the cells were rinsed with PBS to remove residual DCFH-DA. Fluorescence images were captured using a fluorescence microscope, and ImageJ software (National Institutes of Health, Bethesda, MD, USA) was employed to quantify the relative mean fluorescence intensity.

### MitoSOX assay

The MitoSOX assay was carried out using the MitoSOX Assay Kit (S0061S, Beyotime, China). In brief, the preprocessed BMMs were incubated with a 5 μM MitoSOX Red staining solution for 30 min in a 37 °C cell incubator. The cells were then rinsed with PBS to ensure the complete removal of any excess MitoSOX Red. Fluorescence images were captured using a fluorescence microscope.

### TRAP staining

In the TRAP staining procedure, fully differentiated osteoclasts were fixed with 4% paraformaldehyde (PFA). Afterward, the PFA was discarded, and the cells were washed 3 times with PBS buffer. Staining was then performed with the TRAP Kit (G1492, Solarbio, China).

### ALP and ARS staining

ALP and ARS staining were performed following a pre-existing protocol [58]. Briefly, osteoprogenitor cells were cultured under in vitro conditions to undergo osteogenic differentiation for either 7 or 21 days. The cells were then fixed with 4% PFA at room temperature for 15 min. After washing 3 times with PBS, ALP and ARS staining were conducted using the BCIP/NBT Alkaline Phosphatase Color Development Kit (C3206, Beyotime, China) and the ARS Staining Kit (C0148S, Beyotime, China).

### Resorption pit assay

The resorption pit assay was performed according to a previously reported method [59]. In summary, sterilized bovine bone slices were positioned in a 96-well plate, and BMMs were cultured on the slices at a density of 1 × 10^4^ cells per well. A control group without bovine bone slices was included to observe the differentiation process. The BMMs were first induced for osteoclastic differentiation over a period of 5 days to form osteoclasts, followed by treatment with Visomitin for an additional 5 days. Subsequently, the bone slices were collected and analyzed for bone resorption using Zeiss FE-SEM scanning electron microscopy (G300). The resorbed area was measured using ImageJ software. To determine the depth of the resorption pits, osteoclasts from various groups were labeled with iF488-wheat germ agglutinin and imaged along the *Z*-axis using a Nikon A1 Ti confocal microscope for quantification of the pit depths.

### RT-qPCR assay

RNA extraction from cells was carried out using the Ultrapure RNA Kit (#CW0581, CWBIO, China). The extracted mRNA was then reverse-transcribed into cDNA using the Evo M-MLV RT Kit (AG11705, Accurate Biology, China), following the provided instructions. For RT-qPCR analysis, the cDNA was amplified by mixing it with forward primers, reverse primers, and SYBR Green Master Mix (11201ES08, Yeasen, China) in optimized proportions. Quantification was performed on the ABI Prism 7500 System (Applied Biosystems, USA). Internal normalization was performed using the reference gene Actb (β-Actin). The relative expression levels of the specific genes were calculated by employing the 2^−ΔΔCt^ method. A complete list of primer sequences utilized in this study is presented in Table S2.

### WB assay

In the WB analysis, the prepared cells were first lysed with radioimmunoprecipitation assay (RIPA) lysis buffer (FD009, FDBIO, China), which was supplemented with a phosphatase inhibitor cocktail (FD1002, FDBIO, China), 100 mM phenylmethanesulfonyl fluoride (FD0100, FDBIO, China), and a protease inhibitor cocktail (FD1001, FDBIO, China). The mixture was placed on ice for 30 min to ensure thorough cell lysis. Afterward, the lysate was gathered and subjected to centrifugation at 12,000 rpm for 10 min at a temperature of 4 °C. The supernatant, representing the total protein extract, was then harvested and quantified using the BCA Protein Assay Kit (P0012, Beyotime, China).

The extracted protein was separated by electrophoresis on a sodium dodecyl sulfate-polyacrylamide gel electrophoresis gel and subsequently transferred onto a polyvinylidene difluoride (PVDF) membrane using the eBlot L1 Fast Wet Transfer System (L00686C, Genscript, China). Afterward, the blocking of the membrane was performed using 5% skimmed milk at room temperature for a duration of 1 h. The blocked membranes were then incubation with specific primary antibodies at 4 °C overnight. The following day, the PVDF membrane was incubated with the corresponding secondary antibodies at room temperature for 1 h. After each step, the membranes were rinsed 3 times using Tris-buffered saline with Tween 20 (TBST) buffer. Finally, the results were visualized using the eBlot Touch Imager (e-BLOT XLI, Genscript, China).

### Cellular immunofluorescence staining

Cells were plated on sterile cell culture slides at an appropriate density for the experimental treatment. After the designated treatment, the culture medium was removed, and the cells were rinsed with PBS and fixed with 4% PFA. Following fixation, the cells were washed again with PBS and then permeabilized with 1% Triton X-100. Subsequently, the cells were blocked with 5% bovine serum albumin before being incubated with primary antibodies overnight at 4 °C. On the following day, the cells were thoroughly rinsed with PBS to eliminate any unbound primary antibodies and subsequently incubated with fluorescently tagged secondary antibodies for 1 h at room temperature. Afterward, the cells underwent 3 additional washes with PBS to eliminate any remaining secondary antibodies. DAPI (4′,6-diamidino-2-phenylindole) dye was then employed for nuclear staining, and the stained specimens were mounted with an antifade mounting medium. Finally, the slides were subjected to image acquisition and detailed analysis using a fluorescence or confocal microscope.

### Plasmid transfection

pLV2-CMV-STAT3-3×FLAG-Puro plasmids were purchased from Miaoling Biotechnology and validated via DNA sequencing (Tsingke Biotechnology Co., Ltd., China). For transfection experiments, HEK-293T cells were plated at an optimal density and then transfected with Lipofectamine 3000 (#2078159, Invitrogen, USA) following the manufacturer’s instructions. Empty vector plasmids were used as negative controls. Six to eight hours post-transfection, the medium was refreshed, and transfection efficiency was subsequently assessed using WB.

### Adenovirus infection

The LDHB adenovirus utilized in this study was purchased from Genechem. For adenoviral infection, BMMs were initially seeded and cultured at an appropriate density. Subsequently, a mixture of adenovirus and polybrene (H8761, Solarbio, China) at a suitable multiplicity of infection was added for infection. Following an 8-h infection period, the medium was replaced with fresh medium, and infection efficiency was further evaluated through WB and RT-qPCR assays.

### Mitochondrial membrane potential assay

The JC-1 fluorescent probe (C2006, Beyotime, China) was carried out for the mitochondrial membrane potential assay. Specifically, BMMs were cultured in a confocal dish and underwent osteoclastic differentiation in the presence or absence of Visomitin. Subsequently, the 1× JC-1 staining solution was added for cell incubation at 37 °C for a duration of 20 min. Unbound JC-1 probe was then carefully removed by washing before imaging with a Nikon A1 Ti confocal microscope. The relative mean fluorescence intensity was analyzed using ImageJ software to determine the ratio of Aggregate to Monomer.

### Cellular energy metabolism assay

The assessment of cellular energy metabolism was conducted by measuring the oxygen consumption rate (OCR) and extracellular acidification rate (ECAR) using the Seahorse XF Cell Mito Stress Test Kit (103015-100, Agilent, USA) and the Seahorse XF Cell Glycolysis Stress Test Kit (103020-100, Agilent, USA). Before analysis, BMMs that underwent different treatments were trypsinized and seeded onto XF Cell Culture Plates. The cells were then incubated overnight at 37 °C in a humidified incubator with 5% CO_2_. Simultaneously, XF Cartridges were hydrated with Seahorse XF calibrant and incubated overnight at 37 °C in an environment without CO_2_. The assay medium was prepared according to the manufacturer’s instructions, and stimulators such as oligomycin, trifluoromethoxy carbonyl cyanide phenylhydrazone, rotenone and antimycin A, glucose, and 2-deoxy-D-glucose were diluted to their recommended concentrations. Following preparation, the cartridges were inserted into the culture plates, and the medium was exchanged with the aforementioned assay medium. This was followed by incubation at 37 °C in a CO_2_-free environment for 1 h. The analysis of cellular energy metabolism was then performed using the Seahorse XF Extracellular Flux Analyzers. To determine intracellular ATP levels, BMMs treated under varying conditions were evaluated using the ATP Assay Kit (S0027, Beyotime, China) following the manufacturer’s protocols.

### Transcriptome RNA-seq

BMMs were cultured under osteoclastic differentiation conditions for 5 days, either in the presence or in the absence of Visomitin treatment. Subsequently, the samples were sent to Shanghai Majorbio Bio-pharm Biotechnology Co., Ltd. for transcriptome RNA-seq. The transcripts per million reads (TPM) method was utilized to determine the transcript expression levels of each genes. The DEGs were defined as those with |log2FC| > 1, *P* < 0.05, and TPM > 0.5.

For bioinformatics analysis, the Database for Annotation, Visualization, and Integrated Discovery (DAVID) database was used to conduct KEGG and GO enrichment analyses. PaGenBase was employed for cell- and tissue-specific expression analysis. GSEA was performed using OmicStudio tools (https://www.omicstudio.cn/tool). Additionally, Metascape was utilized to generate a network visualization of enriched terms.

### Q300 quantitative metabolomics

BMMs were subjected to osteoclastic differentiation for 5 days, with or without Visomitin treatment, followed by Q300 quantitative metabolomics analysis using mass spectrometry conducted by Cosmos Wisdom. Differentially altered metabolites were defined as those exhibiting a significance level of *P* < 0.05. For bioinformatics analysis, the Metaboanalyst 6.0 database was utilized for SMPDB and KEGG enrichment analyses.

### Homology modeling and molecular docking

The 3-dimensional (3D) structure of Visomitin was retrieved from the PubChem database and then imported into Chem 3D v20.0 software for further structural refinement. The UniProt ID for the target, STAT3, is P40763, with the selected crystallographic structure having a PDB ID of 6ghd for further analysis. A model of protein STAT3 bound to DNA was constructed using AlphaFold3 based on the sequence corresponding to 6ghd, which serves as the principal target structure. Both ligand and target structures were imported into AutoDock Tools v1.5.7 software to perform hydrogenation and charge calculations for molecular docking analysis, revealing that the optimal binding energy between ligand Visomitin and STAT3 is −6.956 kcal/mol. The amino acid residues participating in hydrogen bond connections between STAT3 and Visomitin were examined using PyMOL v2.5.4 to create a 3D interaction map: orange double helices represent DNA while yellow stick models depict Visomitin; brown spheres indicate Br^−^ ions; blue cartoon representations illustrate chain A of STAT3 while purple cartoons denote chain B. Furthermore, key amino acid residues within the optimal binding region between ligand Visomitin and protein STAT3 include THR-641 (A), GLN-643 (A), GLN-644 (A), PRO-715 (A), ASN-646 (B), ASN-647 (B), GLU-652 (B), ARG-688 (B), PRO-689 (B), GLU-690 (B), SER-691 (B), LYS-707 (B), THR708 (B), LYS709 (B), and PHE710 (B). Among these residues, THR641 (A) and ASN646 (B) are critical for establishing hydrogen bond interactions with Stat3. For visualization purposes, cartoon models were employed to display protein structures while stick models represented ligands along with their interacting amino acid residues, with yellow dashed lines indicating positions of hydrogen bonds.

### Cellular thermal shift assay

The FLAG-STAT3 transfected HEK-293T cells were exposed to either 300 nM Visomitin or PBS (as a control) for 24 h. Following treatment, the cells were rinsed and collected in PBS supplemented with a protease inhibitor cocktail (P1006, Beyotime, China). Each cell mixture was divided into 6 equal parts and heated at the indicated temperatures (from 45 to 65 °C) for 3 min. After heating, the samples were allowed to stand at room temperature for an additional 3 min before being snap-frozen in liquid nitrogen. Following 2 freeze–thaw cycles and centrifugation, the supernatants were harvested and mixed with loading buffer for subsequent WB assay to detect the level of FLAG-STAT3.

### DARTS assay

HEK-293T cells were lysed using RIPA buffer. The resulting protein extracts were subsequently diluted in TNC buffer (50 mmol/l Tris-HCl, pH 8.0; 50 mmol/l NaCl; 10 mmol/l CaCl_2_) and incubated with Visomitin at concentrations of 0, 75, 150, and 300 nm for 1 h. Subsequently, the protein samples were incubated with 40 ng/ml pronase for 15 min at ambient temperature. The reaction was terminated by the addition of loading buffer, followed by WB assay to assess the expression level of the target protein.

### Chromatin immunoprecipitation assay

RANKL-induced BMMs, treated with or without Visomitin, were fixed with 1% formaldehyde for 10 min. An anti-Stat3 antibody along with the ChIP (chromatin immunoprecipitation) Kit (#9003, Cell Signaling Technology, USA) were employed for the ChIP assay. A species-matched anti-IgG antibody was carried out as a negative control. The DNA fragments enriched in the immunoprecipitates were analyzed by DNA gel electrophoresis using specific primers provided in Table S2.

### Calvarial osteolysis model

The calvarial osteolysis model was developed according to a previously reported method [60]. Specifically, 12-month-old male mice were randomly allocated into experimental groups and anesthetized with 30 mg/kg pentobarbital through intraperitoneal injection. Subsequently, a calvarial osteolysis model was established by subcutaneously injecting 25 mg/kg of LPS (HY-D1056, MedChemExpress, USA). The sham surgery closely mirrored the aforementioned procedure but excluded LPS administration. PBS or varying concentrations of Visomitin were administered starting 2 days post-surgery. Seven days after the surgical intervention, crania were harvested for subsequent experiments.

### OVX model

The methodology for establishing the mouse OVX model is consistent with that described in our previous study [56]. Briefly, 12-month-old female mice were randomly assigned to groups and anesthetized with 30 mg/kg pentobarbital via intraperitoneal injection before being positioned in a prone orientation. An incision was made to excise both ovaries; subsequently, hemostasis was achieved by clamping any bleeding vessels, followed by disinfection of the incisions and suturing. The sham surgery closely mirrored the OVX procedure but did not involve ovary removal. Four weeks post-surgery, PBS or different concentrations of Visomitin were administered, after which the mice were euthanized and their bone tissues were collected for subsequent experiments following another 4-week period.

### Serum biochemical analysis

Before euthanizing SHAM and OVX mice, blood samples were obtained. The whole blood was left to clot at room temperature for 30 min and then centrifuged at 3,000 rpm for 10 min to separate the mouse serum. The serum samples were utilized for enzyme-linked immunosorbent assay (ELISA) and biochemical hepatorenal function detection. For ELISA, the levels of CTXI and P1NP in the serum were measured using ELISA kits (E-EL-M3023/E-EL-M0233, Elabiscience, China). The biochemical hepatorenal function detection was performed by HaoKe Biotechnology.

### Micro-CT scanning

The skull and femur samples were securely fixed in 4% PFA and then subjected to micro-CT scanning (SkyScan 1275; Bruker microCT, Kontich, Belgium) using the following settings: isotropic resolution of 9 μm, x-ray energy configured at 60 kV and 60 mA. The collected data were analyzed with DataViewer software (Version 1.5.6.2) and CTan software (Version 1.20.8.0) to evaluate bone morphometric parameters quantitatively. Lastly, a representative 3D reconstructed image was created using CTvox software (Version 3.3).

### Histological staining

The mice subjected to modeling were euthanized to obtain their bone tissue and organs. For paraffin sectioning, the samples were fixed in 4% PFA for 48 h and subsequently transferred to an EDTA decalcification solution for complete decalcification. The samples were subsequently embedded and sectioned for further staining procedures. In contrast, for cryosectioning, the harvested bone tissue was fixed overnight in 4% PFA and decalcified thoroughly at 4 °C. The specimens were then immersed in CPT solution (20% sucrose and 2% polyvinylpyrrolidone dissolved in PBS) overnight, followed by embedding in optimal cutting temperature (OCT) compound and cryosectioning for specific staining.

For HE staining, TRAP staining, and immunofluorescence, paraffin sections were initially processed for dewaxing and rehydration.. The HE Staining Kit (C0105M, Beyotime, China), TRAP Staining Kit (AMK1005, Amizona Scientific, China), and Immunofluorescence Staining Kit (P0176/P0179, Beyotime, China) were used to stain the sections. Upon completing the staining process, bright-field sections were gradually dehydrated and mounted with neutral resin, while immunofluorescence sections were sealed with an antifade mounting medium.

For DHE staining, a working solution of DHE dye was prepared at a concentration of 10 μM. Cryosections were incubated with this solution for 60 min, followed by thorough washing with PBS before being mounted with an antifade mounting medium.

All sections were imaged using a KFBIO scanning and analysis system (KFBIO, Zhejiang, China), and quantitative analysis was performed using ImageJ software.

### Calcein double labeling

Ovariectomized mice were subjected to peritoneal injections of calcein twice, with each injection administered at a dosage of 10 μg/g body weight on the 10th and 3rd days prior to euthanasia. Following this procedure, femoral specimens were harvested and fixed in 75% ethanol. Subsequently, the samples were processed into non-decalcified hard tissue sections using the EXAKT 310CP Precision Hard Tissue Sectioning System according to the manufacturer’s instructions. The MAR calculation formula is defined as the ratio of the width of 2 calcein deposition lines to the interval between injections (measured in days).

### Statistical analysis

The aforementioned assays included a minimum of 3 biological replicates. The Prism 9 software (GraphPad Software, Inc., San Diego, CA, USA) was carried out for statistical analyses. For comparisons between 2 groups, the Student’s *t* test was applied, while 1-way or 2-way analysis of variance followed by Tukey’s post hoc test was applied for multi-group comparisons. A *P* value < 0.05 was considered statistically significant, with *P < 0.05, **P < 0.01, and ***P < 0.001 indicating varying degrees of significance.

## Data Availability

All data pertaining to this study are provided within the paper or the Supplementary Materials. Requests for any additional data related to this study may be directed to the authors.
