# Supplementary material for: Visomitin Attenuates Pathological Bone Loss by Reprogramming Osteoclast Metabolism via the STAT3/LDHB Axis
Source: Research (Wash D C). 2025 Jul 22;8:0784. doi: 10.34133/research.0784 (PMC12280330; doi:10.34133/research.0784)
Supplement: Supplementary 1 — Figs. S1 to S7 Tables S1 and S2 [file research.0784.f1.zip › Figure Legends.docx]

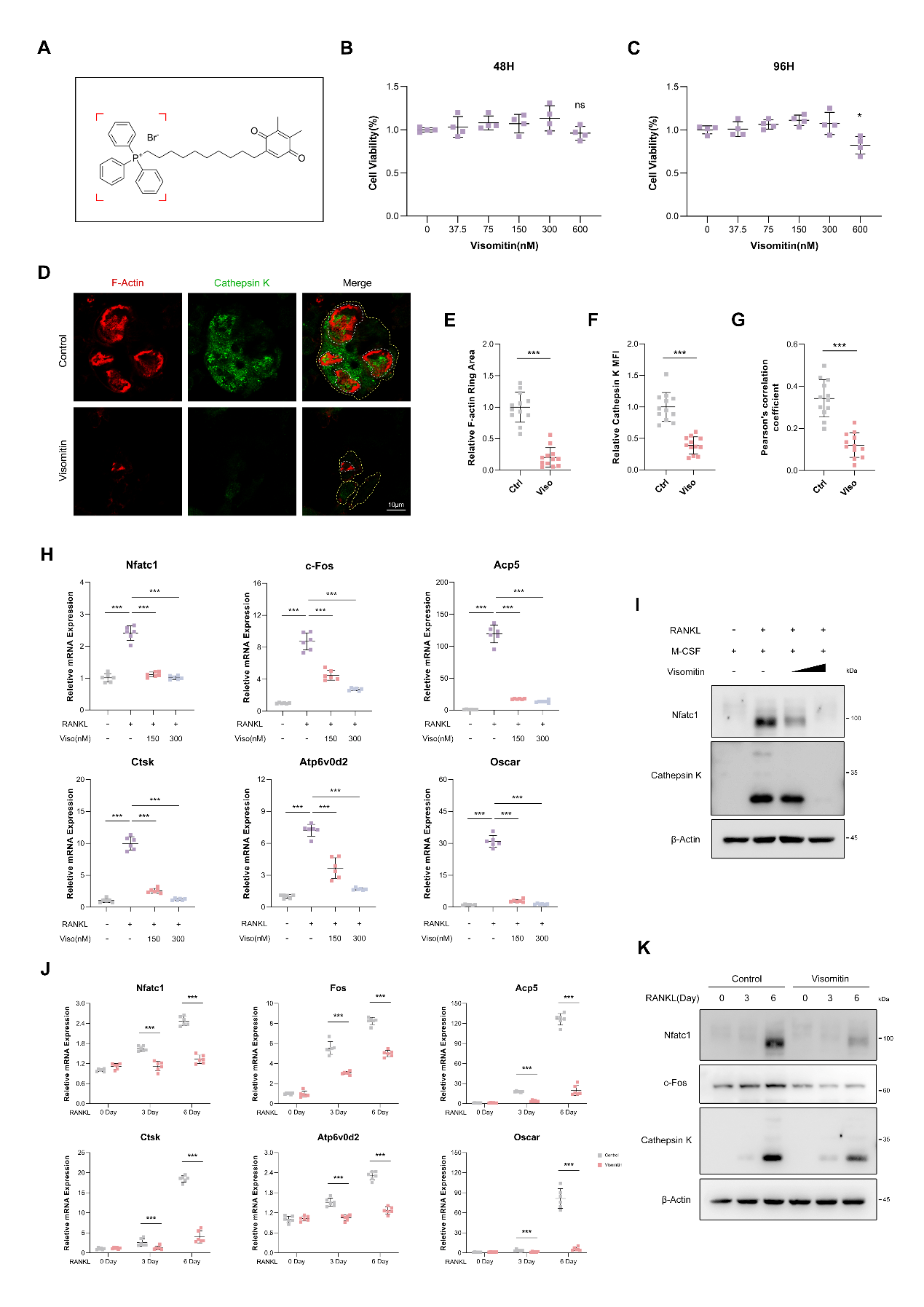


**Supplementary Figure 1. Visomitin mitigates osteoclastogenesis and the expression levels of osteoclast-specific markers.**

A. Representing the chemical structure of Visomitin.

B, C. The cell viability of BMMs exposed to different concentrations of Visomitin for 48 or 96 hours (n = 4).

D. Representative immunofluorescent images of F-Actin and Cathepsin K (CTSK). Scale bars, 10μm.

E, F, G. Quantification of the Relative F-Actin Ring Area, Relative Cathepsin K MFI, and Pearson’s correlation coefficient between F-Actin and Cathepsin K in panel (E) (n=12).

H. The mRNA levels of genes associated with osteoclast differentiation in response to increasing doses of Visomitin were assessed using qRT-PCR (n=6).

I. Representative immunoblots of osteoclast-associated proteins following treatment with increasing doses of Visomitin (n = 3).

J. The mRNA levels of genes implicated in osteoclast differentiation at designated time points, with or without Visomitin treatment, were assessed using qRT-PCR (n=6).

K. Representative immunoblots of osteoclast-associated proteins at designated time points, with or without Visomitin treatment (n = 3).

Data are mean ±SD; *p < 0.05, **p < 0.01, and ***p < 0.001; ns, not significant.


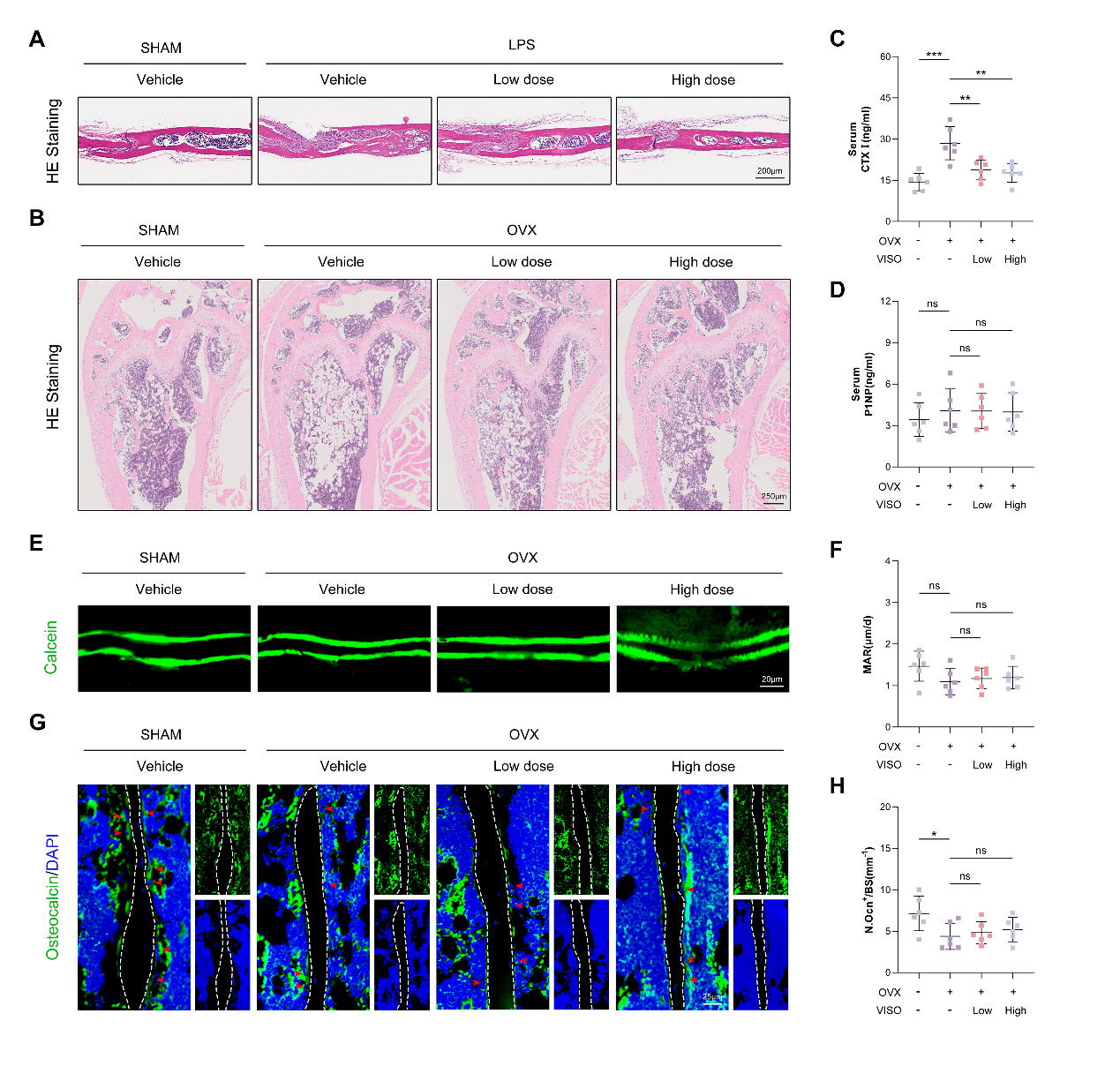


**Supplementary Figure 2.** **Administration of Visomitin exerts slight impact on osteogenesis in vivo.**

A. Representative HE staining of the calvaria from designated groups. Scale bars, 200μm.

B. Representative HE staining of the femur from designated groups. Scale bars, 250μm.

C, D. Quantification of CTXⅠ and P1NP concentrations in the serum of mice from designated groups using ELISA (n=6).

E. Representative images of calcein double labeling in murine femur sections from designated groups; Scale bars, 20μm.

F. Quantification of the Mineral Apposition Rate (MAR) based on calcein double labeling in panel (I) (n=6).

G. Representative immunofluorescence images of osteocalcin in murine femur sections from designated groups; Scale bars, 25μm.

H. Quantification of the N.Ocn^+^/BS(mm^-1^) in panel (G) (n=6).

I. Representative HE staining of organs from OVX mice treated with vehicle and varying dosages of Visomitin; Scale bars, 200μm.

Data are mean ±SD; *p < 0.05, **p < 0.01, and ***p < 0.001; ns, not significant.

**
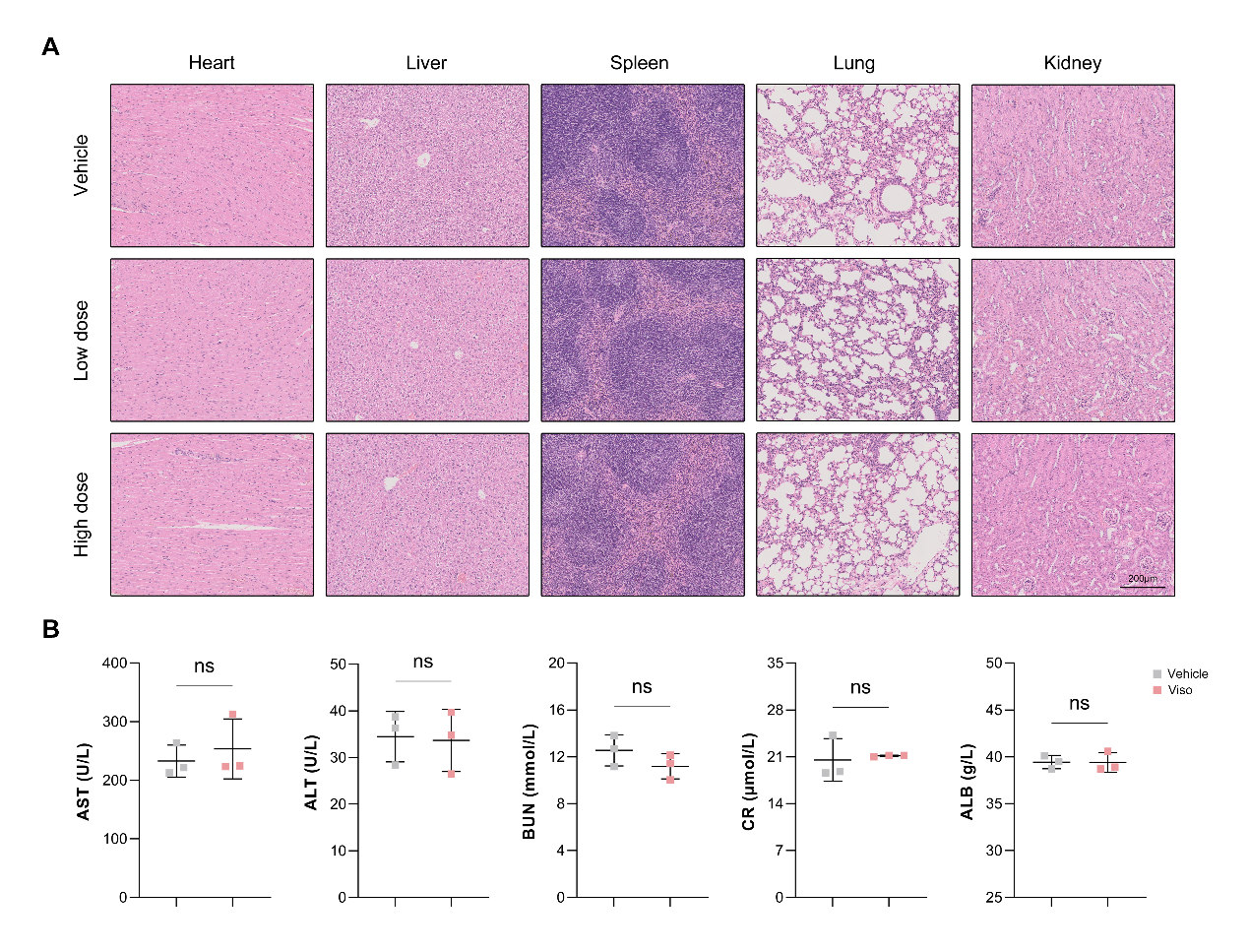
**

**Supplementary Figure 3.** **Evaluation of the In Vivo Safety Profile of Visomitin.**

A. Representative HE staining of organs from OVX mice treated with vehicle and varying dosages of Visomitin; Scale bars, 200μm.

B. Evaluation of biochemical hepatorenal function in mice undergoing different treatments (n=3).

Data are mean ±SD; *p < 0.05, **p < 0.01, and ***p < 0.001; ns, not significant.

**
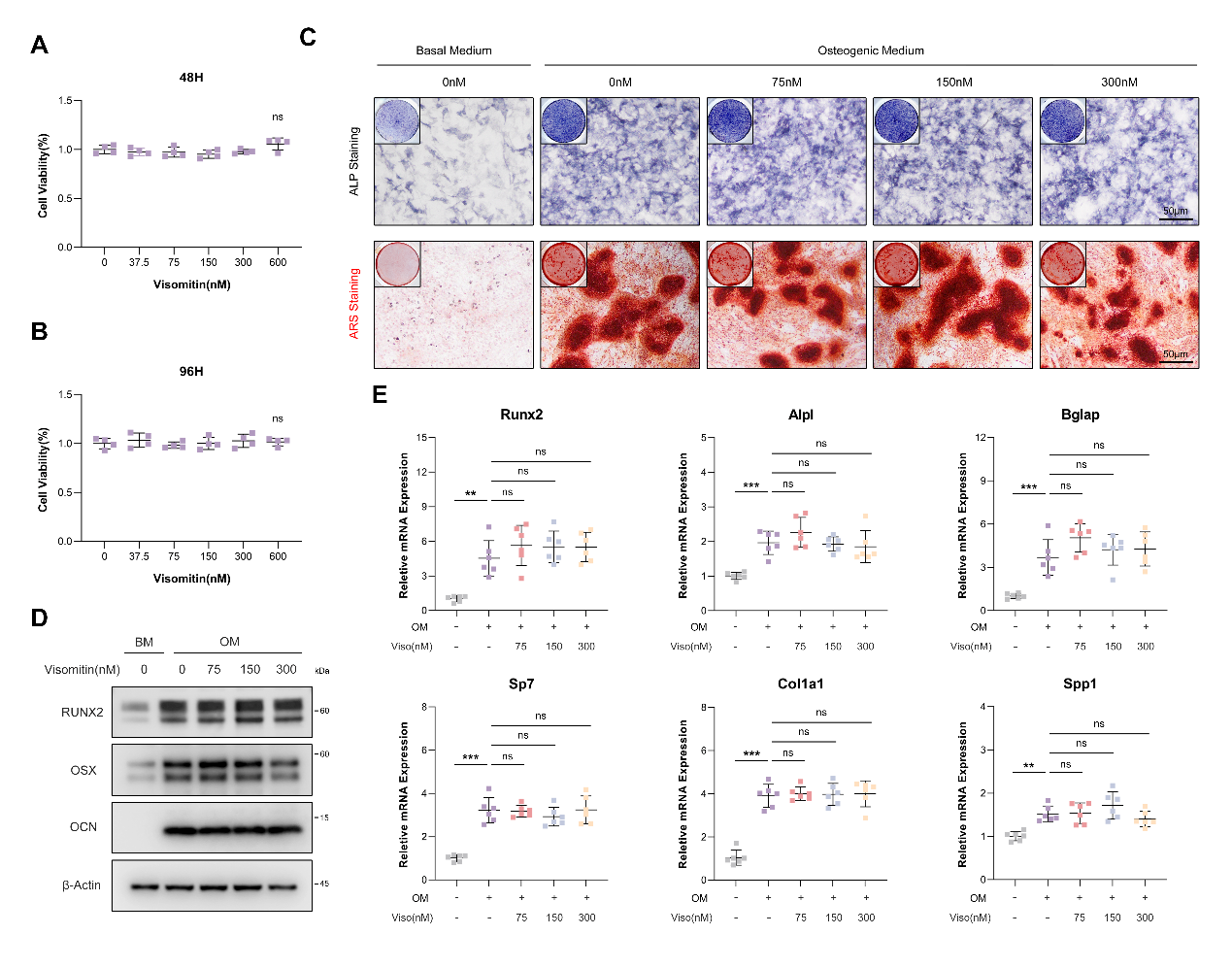
**

**Supplementary Figure 4. Visomitin exerts slight impacts on osteogenesis in vitro.**

A, B. The cell viability of osteoprogenitor cells exposed to different concentrations of Visomitin for 48 or 96 hours (n = 4).

C. Representative images of ALP and ARS staining in osteoblasts exposed to 7 or 21 days of osteogenic differention, with or without Visomitin treatment; Scale bars, 50μm.

D. The mRNA levels of genes implicated in osteogenesis in response to increasing doses of Visomitin were assessed using qRT-PCR (n=6).

E. Representative immunoblots of osteogenic-associated proteins in response to escalating doses of Visomitin.

Data are mean ±SD; *p < 0.05, **p < 0.01, and ***p < 0.001; ns, not significant.

**
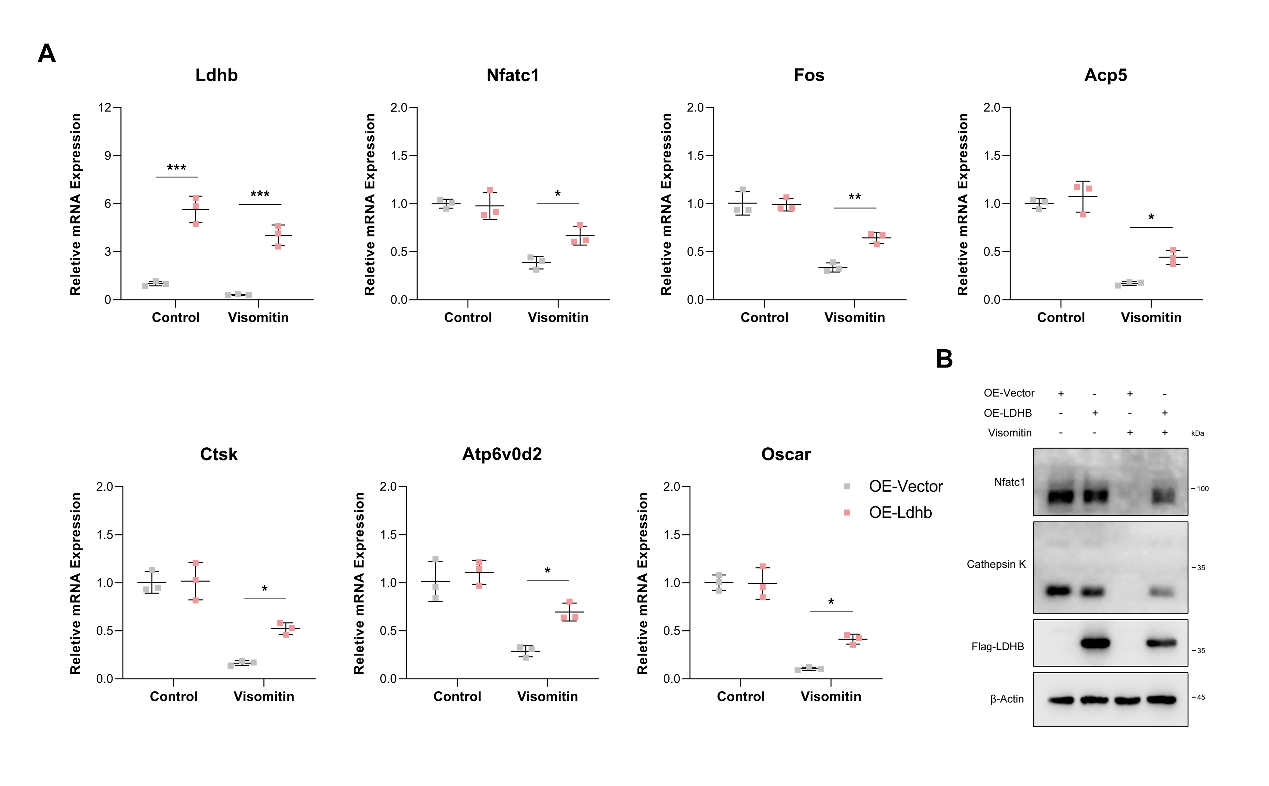
**

**Supplementary Figure 5. Overexpression of LDHB partially reversed the inhibitory effect of Visomitin on osteoclasts.**

A. The mRNA levels of genes associated with osteoclast differentiation in response to LDHB overexpression, with or without Visomitin treatment, were evaluated using qRT-PCR (n=3).

B. Representative immunoblots of osteoclast-associated proteins in response to LDHB overexpression, with or without Visomitin treatment (n = 3).

Data are mean ±SD; *p < 0.05, **p < 0.01, and ***p < 0.001; ns, not significant.

**
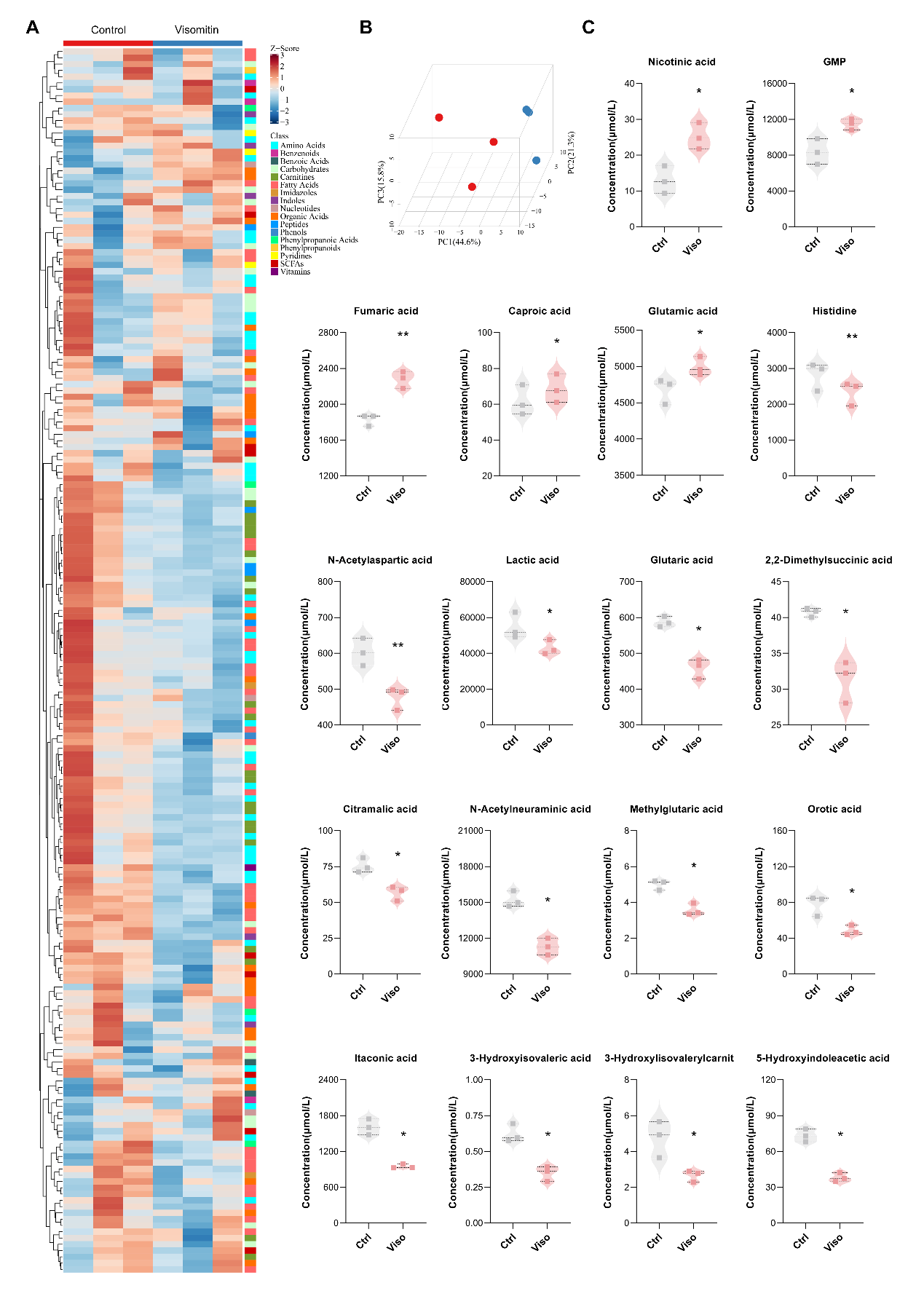
**

**Supplementary Figure 6. Visomitin modulates the metabolite profiles during osteoclastogenesis.**

BMMs treated with Visomitin or PBS were subjected to osteoclast differentiation, followed by Q300^TM^ quantitative metabolomics analysis (n=3).

A. The heatmap illustrating the metabolite profiles derived from Metabolomics.

B. Principal Component Analysis (PCA) of data derived from Metabolomics.

C. The concentration levels of differential metabolites identified through Metabolomics.

Data are mean ±SD; *p < 0.05, **p < 0.01, and ***p < 0.001; ns, not significant.


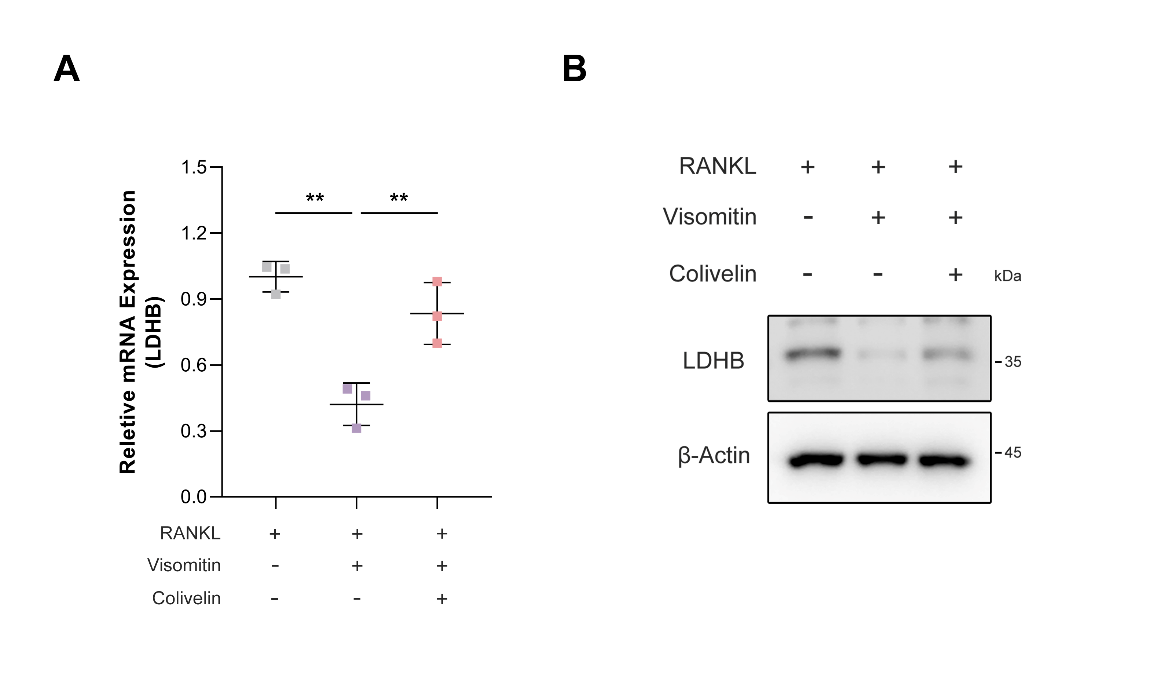


**Supplementary Figure 7. STAT3 functions as a direct ligand of Visomitin to regulate the transcription of LDHB.**

A. The mRNA levels of LDHB in response to Colivelin or Visomitin treatment, as indicated, were assessed using qRT-PCR (n=3).

B. Representative immunoblots of LDHB in response to treatment with Colivelin or Visomitin, as indicated (n = 3).

Data are mean ±SD; *p < 0.05, **p < 0.01, and ***p < 0.001; ns, not significant.
