# Supplementary material for: Visomitin Attenuates Pathological Bone Loss by Reprogramming Osteoclast Metabolism via the STAT3/LDHB Axis
Source: Research (Wash D C). 2025 Jul 22;8:0784. doi: 10.34133/research.0784 (PMC12280330; doi:10.34133/research.0784)
Supplement: Supplementary 1 — Figs. S1 to S7 Tables S1 and S2 [file research.0784.f1.zip › TABLE S1.docx]

| Reagent or Resorce |  |  |
| --- | --- | --- |
| Antibodies | Manufacturer | Catalogue Number |
| Anti-NFATc1 | Santa Cruz | sc-7294 |
| Anti-Cathepsin K | Proteintech | 11239-1-AP |
| Anti-c-Fos | Huabio | ET1701-95 |
| Anti-β-Actin | Proteintech | 66009-1-Ig |
| Anti-p-P65 | Cell Signaling Technology | 3033 |
| Anti-P65 | Cell Signaling Technology | 8242 |
| Anti-p-IkBα | Cell Signaling Technology | 2859 |
| Anti-IkBα | Cell Signaling Technology | 4814 |
| Anti-p-JNK | Cell Signaling Technology | 4668 |
| Anti-JNK | Cell Signaling Technology | 9252 |
| Anti-p-ERK | Cell Signaling Technology | 4370 |
| Anti-ERK | Cell Signaling Technology | 4695 |
| Anti-p-P38 | Cell Signaling Technology | 4511 |
| Anti-P38 | Cell Signaling Technology | 8690 |
| Anti-p-AKT | Cell Signaling Technology | 4060 |
| Anti-AKT | Cell Signaling Technology | 4691 |
| Anti-IDH2 | Huabio | ET1704-93 |
| Anti-IDH3A | Huabio | ER65548 |
| Anti-IDH3B | Proteintech | 68199-1-Ig |
| Anti-Citrate synthetase | Huabio | ET1706-40 |
| Anti-OxPhos Cocktail | Thermofish | 45-8099 |
| Anti-LDHA | Proteintech | 19987-1-AP |
| Anti-LDHB | Huabio | 0807-1 |
| Anti-Stat3 | Cell Signaling Technology | 12640 |
| Anti-p-Stat3 (Tyr705) | Cell Signaling Technology | 9145 |
| Anti-p-Stat3 (Ser727) | Cell Signaling Technology | 9134 |
| Anti-DYKDDDDK/Flag | Yeasen | 30505ES60 |
| Anti-Runx2 | Cell Signaling Technology | 12556 |
| Anti-Sp7 / Osterix | Abcam | ab209484 |
| Anti-Osteoclacin | Huabio | ER1919-20 |
| Anti-IgG Isotype Control | Cell Signaling Technology | 3900 |
| Goat anti-Rabbit Alexa Fluor™ Plus 488 | Thermofish | A32731 |
| Goat anti-Rabbit Alexa Fluor™ Plus 594 | Thermofish | A32740 |

**Supplementary Table.1**
