# Supplementary material for: Visomitin Attenuates Pathological Bone Loss by Reprogramming Osteoclast Metabolism via the STAT3/LDHB Axis
Source: Research (Wash D C). 2025 Jul 22;8:0784. doi: 10.34133/research.0784 (PMC12280330; doi:10.34133/research.0784)
Supplement: Supplementary 1 — Figs. S1 to S7 Tables S1 and S2 [file research.0784.f1.zip › TABLE S2.docx]

**Supplementary Table.2**

| RT-qPCR / ChIP Primer Sequence | |  |  |
| --- | --- | --- | --- |
| Gene | Forward | | Reverse |
| Mouse β-Actin | GGCTGTATTCCCCTCCATCG | | CCAGTTGGTAACAATGCCATGT |
| Mouse Nfatc1 | CAAGTTTCCACTCGGCCCTC | | GAGTGCTATCGGTGGTCAGG |
| Mouse Fos | TTTCAACGCCGACTACGAGG | | GTAAGTAGTGCAGCCCGGA |
| Mouse Acp5 | AAGAGATCGCCAGAACCGTG | | ACTCAGCACATAGCCCACAC |
| Mouse Ctsk | GAGGAAATGCTGGACACCCA | | TTCAGGGCTTTCTCGTTCCC |
| Mouse Atp6v0d2 | CAGAGCTGTACTTCAATGTGGAC | | AGGTCTCACACTGCACTAGGT |
| Mouse Oscar | CCTAGCCTCATACCCCCAG | | CGTTGATCCCAGGAGTCACAA |
| Mouse Ldhb | CATTGCGTCCGTTGCAGATG | | GGAGGAACAAGCTCCCGTG |
| Mouse Runx2 | AGAGTCAGATTACAGATCCCAGG | | TGGCTCTTCTTACTGAGAGAGG |
| Mouse Alpl | CCAACTCTTTTGTGCCAGAGA | | GGCTACATTGGTGTTGAGCTTTT |
| Mouse Bglap | GTATGGCTTGAAGACCGCCT | | GACAGGGAGGATCAAGTCCC |
| Mouse Sp7 | ATGGCGTCCTCTCTGCTTG | | TGAAAGGTCAGCGTATGGCTT |
| Mouse Col1a1 | GCTCCTCTTAGGGGCCACT | | CCACGTCTCACCATTGGGG |
| Mouse Spp1 | AGCAAGAAACTCTTCCAAGCAA | | GTGAGATTCGTCAGATTCATCCG |
| Mouse Ldhb for ChIP | GCGACTCCTAGGGAAGTTACA | | AGCTGCCCAACAAGATCGAA |
